# Burden of colorectal cancer and its risk factors in the North Africa and Middle East (NAME) region, 1990–2019: a systematic analysis of the global burden of disease study

**DOI:** 10.1186/s12889-024-18027-6

**Published:** 2024-02-22

**Authors:** Fahimeh Haghighatdoost, Kamran Mehrabani-Zeinabad, Parisa Hajihashemi, Noushin Mohammadifard, Peyman Adibi

**Affiliations:** 1 Isfahan Cardiovascular Research Center, Cardiovascular Research Institute, Isfahan University of Medical Sciences, Isfahan, Iran; 2 Isfahan Gastroenterology and Hepatology Research Center, Isfahan University of Medical Sciences, Isfahan, Iran

**Keywords:** Global burden of disease, Lifestyle and metabolic risk factors, Colorectal cancer, Disability adjusted life years, North Africa and Middle East

## Abstract

**Objective:**

The geographical differences in incidence rates of colorectal cancer (CRC) and its burden due to modifiable risk factors warrant investigating the CRC burden and its risk factors in different regions. In the current study, we aimed to estimate the burden of CRC and the share of its risk factors in the North Africa and Middle East (NAME), from 1990 to 2019.

**Study design:**

Systematic review.

**Methods:**

The rates of incidence, prevalence, death, years of life lost (YLL), years lived with disability (YLD), and disability adjusted life years (DALYs) of CRC were estimated through the framework of the Global Burden of Diseases (GBD), Injuries and Risk Factors Study 2019 by age, sex, between 1990 and 2019. The CRC-related DALYs attributable to each lifestyle and metabolic risk factor was also estimated through a comparative risk assessment approach.

**Results:**

In NAME region, the trends of incidence, prevalence, death, YLL, YLD, and DALYs of CRC were increasing, with higher rates in males than females over this period. High and high-middle socio-demographic index (SDI) countries had greater CRC DALYs rate compared with middle- and low-SDI countries in 2019, except for Palestine [434.66 (95% UI: 368.82, 503.88)]. In NAME region, like the global, dietary risk (33.18%), low whole grain intake (19.79%), and low intake of milk (15.77%) were the major contributing risk factors to DALYs due to CRC in 2019.

**Conclusions:**

Due to increasing trend of CRC burden and the considerable role of lifestyle and metabolic factors in its burden in NAME region, implementing fundamental strategies to minimize CRC burden and its risk factors is imperative.

**Supplementary Information:**

The online version contains supplementary material available at 10.1186/s12889-024-18027-6.

## Introduction

Epidemiologic transition accounts for the replacement of the communicable infectious diseases by chronic non-communicable diseases (NCD) across the world and noticeably in developing countries [1, 2]. Currently, cancer has been identified as a major contributor to global disease burden and death [3, 4]. The Global Burden of Diseases (GBD), Injuries, and Risk Factors Study 2019 showed that colorectal cancer (CRC) constitutes the third leading cause of cancer deaths and the second leading cause of disability adjusted life years (DALYs) for cancer worldwide [5]. CRC is associated with lower health-related quality of life, functional status, and increased economic burden [6]. 

The relevance of non-modifiable risk factors such as genetic susceptibility, a personal history of polyps or adenoma, or a family history of CRC in the pathogenesis of CRC has been well-established [7, 8]. Furthermore, modifiable risk factors such as unhealthy diet, smoking, alcohol use, physical inactivity, high fasting plasma glucose (HPG), and high body mass index (BMI) play crucial role in the pathogenesis and progression of CRC [9, 10]. Recent evidence suggests that lifestyle risk factors influence the risk of CRC through a variety of mechanisms, including immune dysfunction, inflammation, and gut microbiota alteration [9–11]. As a result, there is great potential to reduce the burden of CRC by prevention and alleviation of modifiable risk factors.

With respect to differences in the distribution of these risk factors, the incidence and mortality rate of CRC may vary in different countries and regions [9]. Rapid economic and social development in developing nations were concomitant with shift in lifestyle habits and increasing the selection of Western diet (e.g., an unhealthy diet (low in whole grain, fruits and vegetables and high in red and processed meat) [12, 13]. Consequently, the rate of CRC incidence may accelerate in low-income and middle-income countries (LMICs) [5]. 

The alarming rise in incidence rates of CRC in LMICs warrants further work to determine the main risk factors and their burden in different regional and national levels to provide an insight into required interventions to mitigate the prevalence and incidence of the disease. Furthermore, North Africa and Middle East (NAME) comprising neighboring countries share important religion, socio-economic and cultural features but also diverse in health indicators [14, 15] Therefore, tracking regional and national trends and burden of CRC and its risk factors can provide policy makers with an overview regarding the health status of their countries and guide them to implement applicable strategies to prevent and manage the disease.

In the present study, we aimed to estimate the burden of CRC in NAME region and explore the contribution of various lifestyle and metabolic risk factors to the burden of disease. We used the comparative risk assessment framework of the GBD Study to estimate burden of CRC attributable to 12 potentially modifiable lifestyle risk factors from 1990 to 2019, by age group and sex.

## Methods

GBD 2019, coordinated by the Institute of Health Metrics and Evaluation (IHME), provides the most comprehensive source of the burden of diseases in 204 countries and territories from 1990 to 2019. It estimates epidemiological measures such as incidence, prevalence, and death rates as well as health summary measures such as years of life lost (YLL), years lived with disability (YLD), and DALYs; all measures are available by age and sex. YLD refers to the number of years lived with any disability weighted by the severity of the health state, YLL refers to how many years of life were lost due to premature mortality, and DALYs were calculated by summing YLL and YLD. All of the data used in this study were gathered from GBD results tool [16]. Detailed information on GBD 2019 was reported previously [17, 18].

GBD groups the countries into seven super regions, including (1) Central Europe, Eastern Europe, and Central Asia, (2) High-income, (3) Latin America and Caribbean, (4) North Africa and Middle East, (5) South Asia, (6) Southeast Asia, East Asia, and Oceania, (7) Sub-Saharan Africa. NAME region includes 21 countries: Afghanistan, Algeria, Bahrain, Egypt, Iran (Islamic Republic of), Iraq, Jordan, Kuwait, Lebanon, Libya, Morocco, Oman, Palestine, Qatar, Saudi Arabia, Sudan, Syrian Arab Republic, Tunisia, Turkey, United Arab Emirates (UAE), and Yemen.

There are three-tiered classification of causes for deaths and disabilities in the GBD. CRC is categorized as one of the 169 level 3 causes under the non-communicable diseases, specifically within the neoplasms subset. This classification encompasses deaths and disabilities arising from invasive neoplasms of the colon and rectum. Details regarding the constituents of level 1 and level 2 are described in Supplementary Table 1. CRC was diagnosed with the following codes of international classification of diseases, tenth edition (ICD10): C18-C19.0, C20, C21-C21.8, Z12.1-Z12.13, Z85.03-Z85.048, Z86.010.

For CRC, there are six available level 2’s attributed lifestyle and metabolic risk factors: alcohol use, dietary risks, high BMI, HPG, low physical activity, and tobacco. As dietary risks are the most contributing risk factor for CRC, we also investigated the six sub-risks (level 3) of dietary risks including diet high in processed meat, diet high in red meat, diet low in calcium, diet low in fiber, diet low in milk, and diet low in whole grains. We also considered another risk factor as remaining which refers to CRC DALYs related to undetermined and non-modifiable risk factors such as genetic susceptibility. Definition of these risk factors are presented in Tables 1 and 2, respectively.

**Table 1:** Definition of colorectal cancer’s level 2 risks

Level 2 Risks	Definition
Alcohol use	Individuals consuming at least one alcoholic beverage in the past year. The level of exposure was estimated based on average grams of pure alcohol consumed per day.
Dietary risks	An aggregate risk factor for 6 dietary risks: diet low in whole grains, fiber, milk, and calcium; and diet high in red or processed meat. If at least one of the six child risks be positive, the parent dietary risk would be positive.
High body mass index	For adults (ages 20 and older): BMI greater than 25 kg/m^2^. For children (ages 1–19): being overweight or obese based on International Obesity Task Force standards.
High fasting plasma glucose	Serum fasting plasma glucose of greater than 4·8–5·4 mmol/L.
Low physical activity	Average weekly physical activity (at work, home, transport-related, and recreational) of less than 3000–4500 total metabolic equivalents (METs) minutes per week.
Tobacco	Tobacco smoking, chewing tobacco use, and secondhand smoke exposure.

BMI, High body mass index

**Table 2:** Definition of colorectal cancer’s level 3 dietary risks

Level 3 Dietary Risks	Definition
High processed meat	Any intake (in grams per day) of meat preserved by smoking, curing, salting, or addition of chemical preservatives.
High red meat	Any intake (in grams per day) of red meat including beef, pork, lamb, and goat but excluding poultry, fish, eggs, and all processed meats.
Low calcium	Average daily consumption (in grams per day) of less than 1·06–1·10 g of calcium from all sources, including milk, yoghurt, and cheese.
Low fiber	Average daily consumption (in grams per day) of less than 21–22 g of fiber from all sources including fruits, vegetables, grains, legumes, and pulses.
Low milk	Average daily consumption (in grams per day) of less than 360–500 g of milk including non-fat, low-fat, and full-fat milk, excluding soy milk and other plant derivatives.
Low whole grains	Average daily consumption (in grams per day) of less than 140–160 g of whole grains (bran, germ, and endosperm in their natural proportion) from breakfast cereals, bread, rice, pasta, biscuits, muffins, tortillas, pancakes, and other sources.

Age standardization was performed via a direct method, applying the estimated age structure of the global population from 2019 [19]. A 95% uncertainty interval (UI) was reported for each measure which were calculated by repeating all calculations 1000 times and taking the 2.5th and 97.5th percentiles of them.

The Socio-Demographic Index (SDI) is a composite indicator of total fertility rate under 25 years old, average years of schooling, and lag distributed income per capita. We used the reference SDI quantile to classify countries by their SDIs in 2019 into five groups including low, low-middle, middle, high-middle, and high [20]. All analyses performed by using R software, version 4.2.1 [21]. GBD risk factors estimation was performed in a six-step risk assessment framework. Details regarding these steps have been described elsewhere [18]. Briefly, these steps include (1) having convincing or plausible evidence for risk-outcomes association, (2) estimation relative risk (RR) for each risk-outcome pair, (3) distribution of exposure for each risk factor by age, sex, location, and year, (4) the theoretical minimum risk exposure level (TMREL), (5) estimation of the population attributable fraction (PAF) and attributable burden, and (6) estimating the PAF and attributable burden for the combination of risk factors [18, 22]. Amongst a total of 87 risk factors defined by the GBD, 12 risk factors (alcohol use, dietary risk, high BMI, HPG, low physical activity, smoking, diet low in whole grain, diet low in calcium, diet low in milk, diet high in red meat, diet high in processed meat, and diet low in fiber) were identified to be related with CRC and were included in this study.

## Results

All presented rates in the current article are age standardized and per 100,000 populations.

### Overview of CRC burden in NAME region

**Fig. 1:**
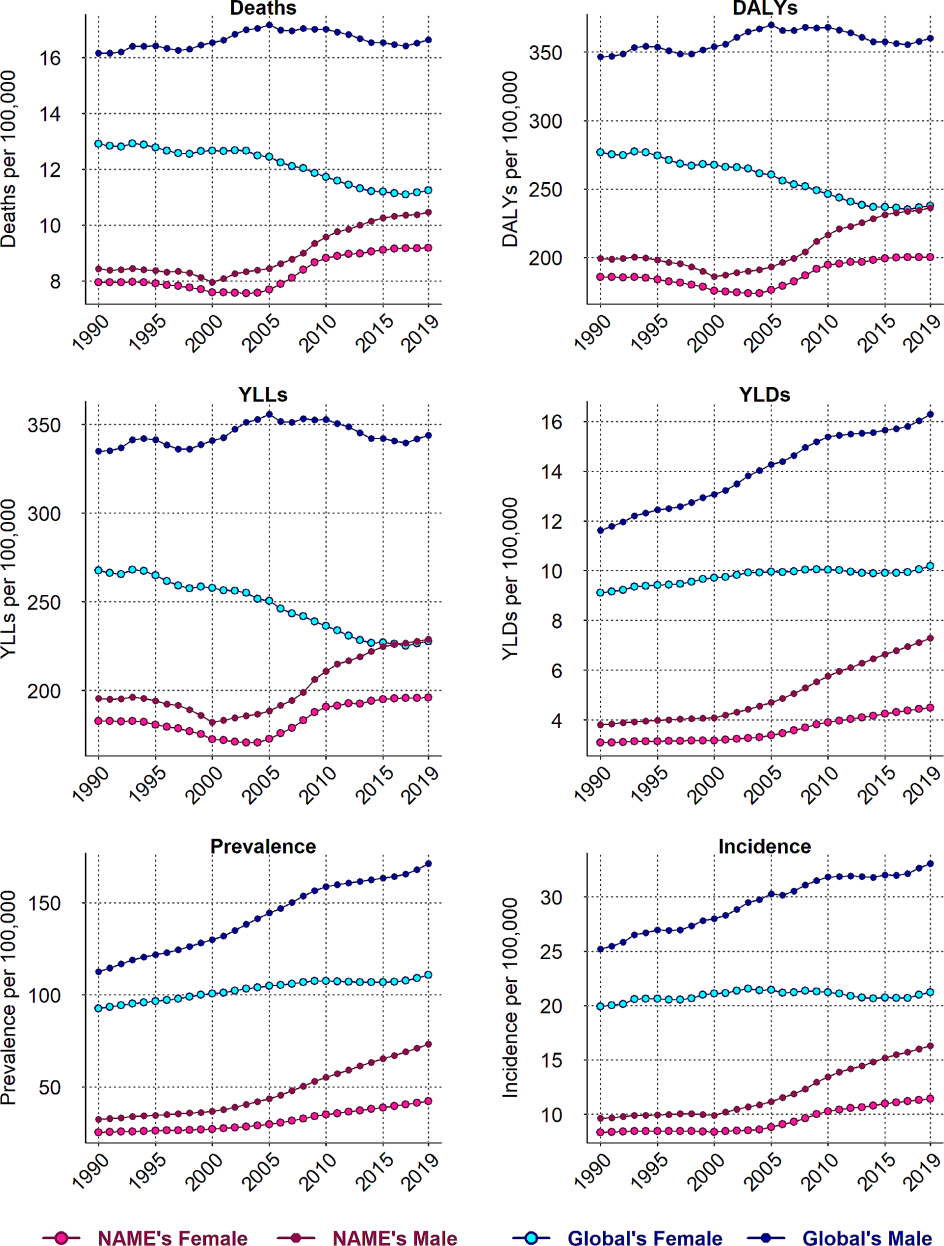
Trends of deaths, DALYs, YLL, YLD, Prevalence and Incidence rates of colorectal cancer in NAME and global region. DALYs, disability adjusted life years; YLL, years of life lost; YLD, years lived with disability; NAME, North Africa and Middle East

Figure 1 and Supplementary Table 2 illustrate the rates of deaths, DALYs, YLL, YLD, prevalence and incidence of CRC in NAME and global region between 1990 and 2019. Overall, in comparison with females, males experienced greater increase in terms of CRC values over study time across NAME region. The incidence rate of CRC increased from 9.64 (95% UI: 7.80, 11.75) to 16.31(95% UI: 14.29, 18.65) in males and from 8.35 (95% UI: 7.08, 9.89) to 11.43 (95% UI: 10.04, 12.96) in females from 1990 to 2019. The growth rate for males and females for the following metrics were respectively: 125.84% % and 65.90% for prevalence, 24.09% and 15.60% for deaths, 18.54% and 7.78% for DALYs, 17.13% and 7.14% for YLL, and 91.17% and 45.65% for YLD. The rates of CRC were much lower in NAME compared with the global in both sexes over the study period. However, unlike NAME region, the global trend of deaths, YLL and DALYs in females was downward during 1990–2019 (-12.97%, -14.92% and − 14.04%, respectively).

### **CRC burden by the GBD super regions**

**Fig. 2:**
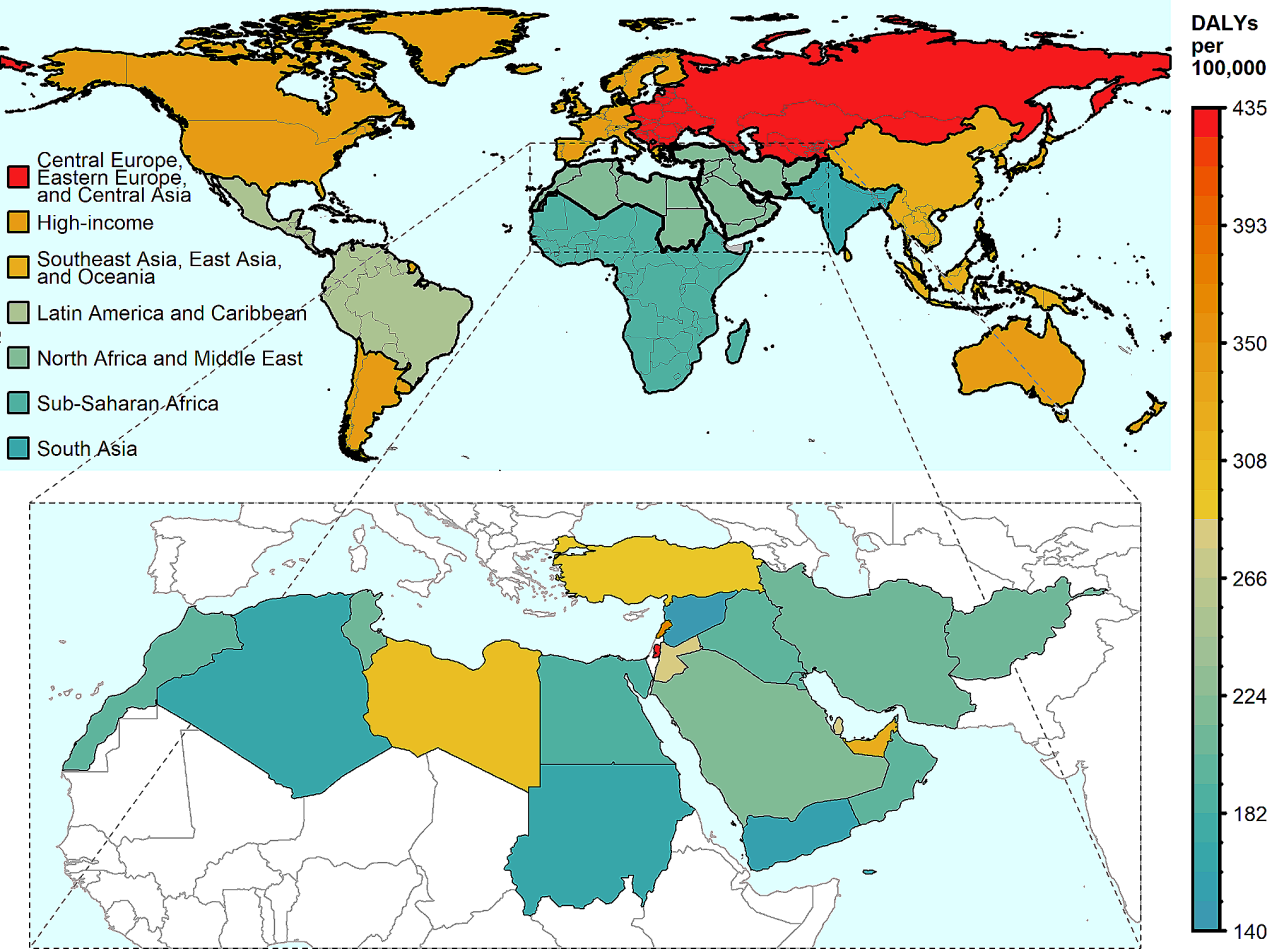
Distribution map of colorectal cancer DALYs in NAME region in 2019 by gender. DALYs, disability adjusted life years; NAME, North Africa and Middle East

Among the GBD super regions, high income [445.81 (95% UI: 432.26, 455.61)] and Central Europe, Eastern Europe, and Central Asia [433.22 (95% UI: 399.15, 469.87)] had the greatest CRC DALYs rates in 1990 and 2019, while south Asia had the lowest DALYs rate [165.06 (95% UI: 141.74, 189.86)] in both 1990 and 2019 **(**Fig. 2 and Supplementary Table 3). From 1990 to 2019, the CRC DALYs trend was increasing in the GBD super regions, except for Central Europe, Eastern Europe, and Central Asia, and high-income regions where it declined by -1.57% and − 21.96%, respectively. NAME with CRC DALYs rate of 218.67 (95% UI: 194.05, 246.53) was considered a low-affected region in 2019. The exceptions were Palestine [434.66 (95% UI: 368.82, 503.88)], Lebanon [372.53 (95% UI: 300.31, 471.78)] and UAE [327.12 (95% UI: 223.85, 452.27)]. Furthermore, the trend of DALYs in NAME region was increasing (13.40%) from 1990 to 2019. The percent change of DALYs varied from − 16% in Bahrain to 47% in Saudi Arabia in the region.

NAME countries can be categorized into six groups based on the similarity in the CRC DALYs in 2019 in comparison with the super regions **(**Fig. 2 and Supplementary Table 3): (1) Syrian Arab Republic, Yemen, Sudan, and Algeria countries were similar to South Asia region; (2) Egypt, Morocco, Iraq, Oman, Kuwait, Iran, Tunisia, and Afghanistan countries were similar to Sub-Saharan Africa region; (3) Saudi Arabia, Bahrain, Jordan, and Qatar countries were similar to Latin America and Caribbean region; (4) Turkey, Libya, and United Arab Emirates countries were similar to Southeast Asia, East Asia, and Oceania region; (5) Lebanon was similar to high-income region; and (6) Palestine was similar to Central Europe, Eastern Europe, and Central Asia region.

### CRC DALYs by countries

High and high-middle SDI countries had greater DALYs rates of CRC compared with middle- and low-SDI countries in 2019. The exception was Palestine, a low-middle SDI country with the highest DALYs rate in the region [434.66 (95% UI: 368.82, 503.88)] **(**Fig. 2 and Supplementary Table 3). Among high SDI countries, UAE had the greatest DALYs rate [327.12 (95% UI: 223.85, 452.27)], and Kuwait had the lowest DALYs rate [202.28 (95% UI: 168.79, 242.15)]. In high-middle SDI, the maximum value of DALYs was observed in Lebanon [173.9 (95% UI: 125.6, 227.1)] and the minimum value belonged to Oman [199.12 (95% UI: 162.35, 243.38)]. Among middle SDI countries, Tunisia had the highest DALYs value [208.08 (95% UI: 151.00, 280.73)], whereas Syrian Arab Republic had the lowest value [140.02 (95% UI: 102.06, 185.69)]. Iran as a middle SDI country with a DALYs rate of [206.73 (95% UI: 192.19, 222.07)] is ranked among countries with low DALYs rate caused by CRC. In the low-middle SDI countries, the highest DALYs rate was in Palestine [434.66 (95% UI: 368.82, 503.88)] and the lowest was in Sudan [164.39 (95% UI: 116.89, 242.57)]. Among low SDI countries, the maximum DALYs rate was seen in Afghanistan [209.28 (95% UI: 137.22, 281.82)], while the minimum rate was seen in Yemen [156.04 (95% UI: 116.18, 211.94)].

From 1990 to 2019, while the DALYs trend was downward in some countries in high and middle- high SDI categories (-9.76% in UAE, -16.83% in Bahrain, -9.34% in Turkey and − 2.45% in Jordan), the trend was constantly upward in all other SDI countries **(**Fig. 2 and Supplementary Table 3).

### CRC-related DALYs attributed to lifestyle and metabolic risk factors

**Fig. 3:**
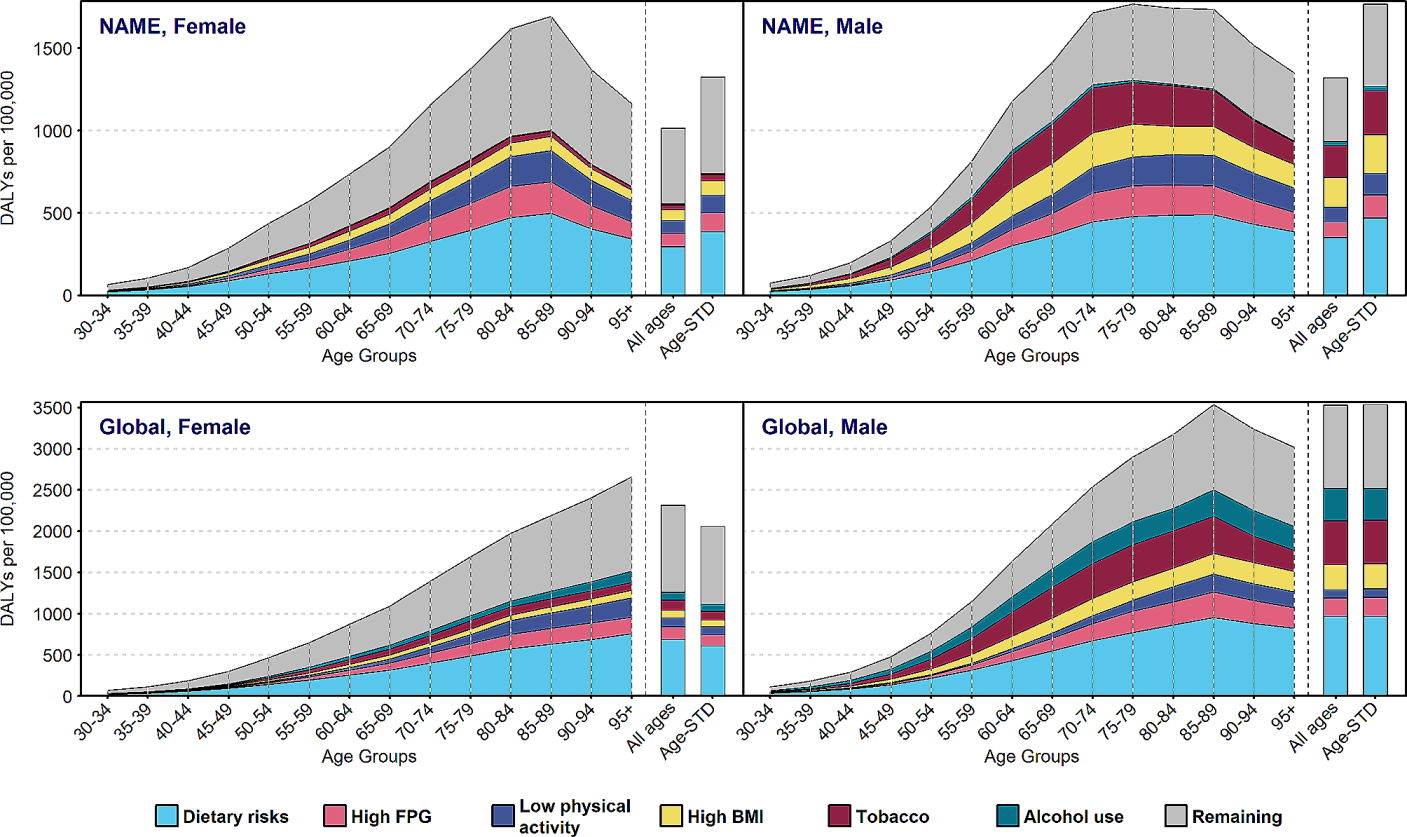
Age trends of colorectal cancer DALYs attributed to risks in NAME and global region in 2019 by gender. DALYs, disability adjusted life years; NAME, North Africa and Middle East; FPG, high fasting plasma glucose

Figure 3 and Supplementary Table 3 show CRC DALYs separately for males and females and age groups according to the six different lifestyle factors and metabolic risk factors in NAME and global region in 2019. In both males and females, either at NAME or at the global level, the major contributor in all age groups was dietary risk. However, the smallest share belonged to alcohol use in NAME and low physical activity globally in both sexes in most of the age groups (Fig. 3 and Supplementary Table 4). Of note, the proportion of risk factors was higher in males than that of in females in most of the age groups. In NAME region, in both sexes, the trend of DALYs attributable to all risk factors, except for alcohol use, for all age groups up to 90 was rising. Notably, females experienced higher decreasing at the age of 90. Contrary to NAME region, the DALYs trend attributed to lifestyle and metabolic risk factors was constantly rising in females at the global level.

### CRC-related DALYs attributable to dietary risk factors

**Fig. 4:**
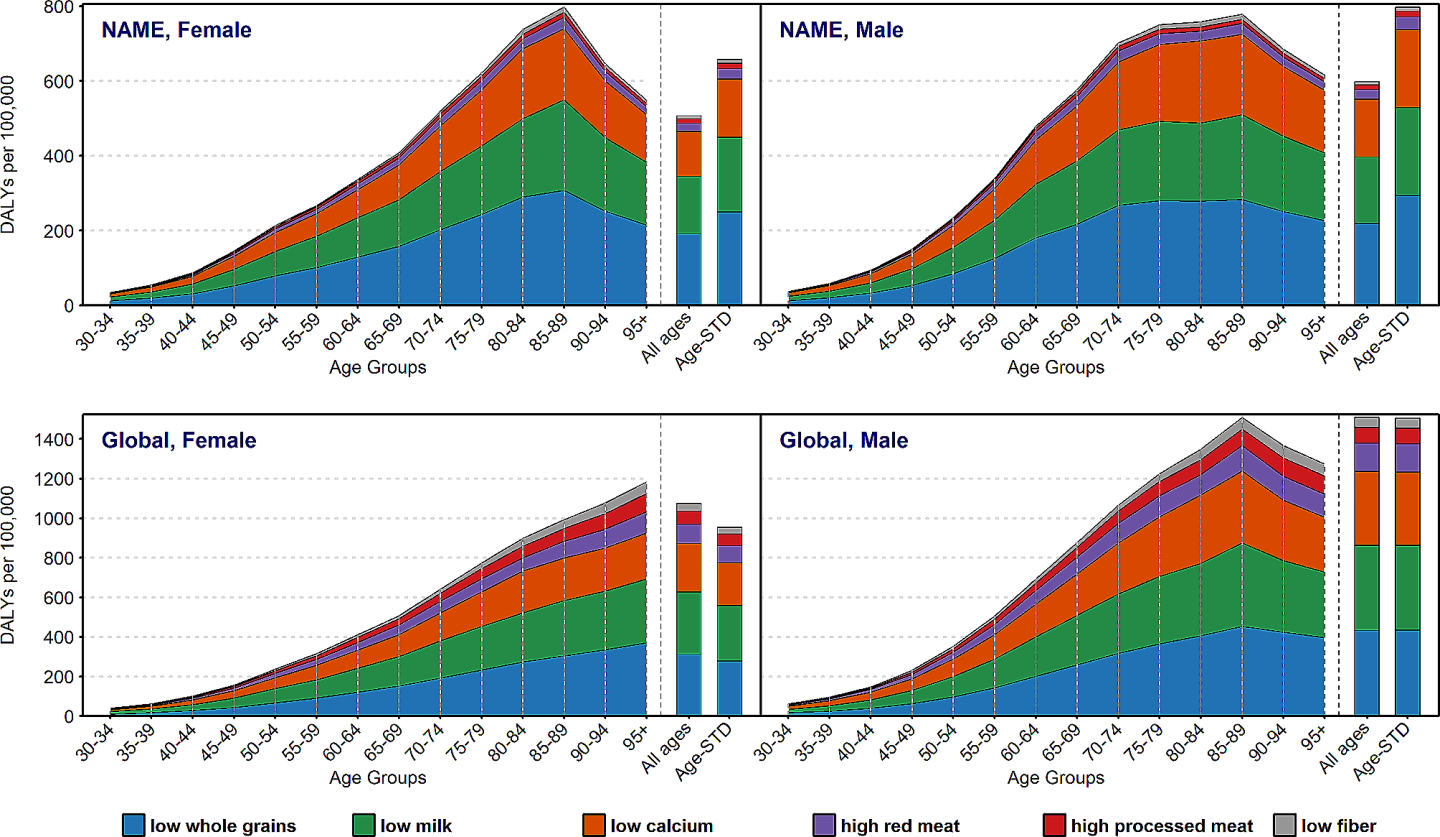
Age trends of colorectal cancer DALYs attributed to dietary risks factors in NAME and global region in 2019 by gender. DALYs, disability adjusted life years; NAME, North Africa and Middle East

Figure 4 and Supplementary Table 5 depict CRC DALYs separately for males and females and age groups according to dietary risk factors in NAME and global region in 2019. In NAME, in both males and females, the low intake of whole grains had the most contribution to CRC DALYs, followed by low intake of milk and low intake of calcium in 2019. The trend was constant across different age groups. By contrast, diets low in fiber and high in possessed meat had the least share in CRC DALYs. Globally, diet low in milk was the main contributor to CRC DALYs up to age group of 60–65 in males and 65–69 in females, after that diet low in whole grain was the main risk factor, whereas low fiber intake had the lowest contribution in all age groups, but not 30–34. However, the DALYs rate of all dietary risk components reduced from the age 90 in NAME region. Unlike NAME, the CRC DALYs contributed to dietary risk factors had a steadily increase in females globally **(**Fig. 4 and Supplementary Table 5).

### CRC-related DALYs attributable to 12 risk factors

Tables 3 and 4, Supplementary Fig. 1 and Supplementary Fig. 2 illustrate the contribution of 12 risk factors to DALYs due to CRC in 2019. Like the global, the main contributing risk factors were dietary risk (33.18%), low whole grain intake (19.79%), and low intake of milk (15.77%). In stratified analysis by sex, the same results were observed, except for smoking (18.95%) which was the third most contributing risk in males. The proportion of each risk factor differed between males and females. For instance, high BMI accounted for 17.0% and smoking accounted for 18·95% of DALYs in males, while the corresponding values in females were 7.33% and 3.05%, respectively.

**Table 3:** Age trends of percentages of colorectal cancer DALYs attributed to risks in NAME and global region in 2019 by gender

Location	Gender	Risk	30-34s	35-39s	40-44s	45-49s	50-54s	55-59s	60-64s	65-69s	70-74s	75-79s	80-84s	85-89s	90-94s	95+	All Ages	Age STD
North Africa and Middle East	Female	Dietary risks	34.77	34.34	33.87	33.72	33.07	32.27	32.03	32.25	32.50	32.82	33.48	33.48	33.30	33.16	32.44	32.51
		High FPG	1.71	2.44	3.43	5.12	7.08	9.08	11.02	12.43	13.29	13.67	13.45	12.88	11.75	10.17	8.92	9.85
		Low phys. act.	5.25	5.39	5.72	6.22	6.72	7.42	8.69	10.03	11.15	12.01	12.62	12.58	12.51	12.52	8.37	9.03
		High BMI	6.00	6.70	7.37	7.76	8.21	8.30	8.18	7.84	7.36	6.74	5.95	6.00	6.04	6.07	7.39	7.33
		Tobacco	0.42	0.91	1.68	2.07	3.09	3.94	4.10	4.27	3.77	2.93	2.63	1.94	1.81	1.70	2.94	3.05
		Alcohol use	0.66	0.61	0.73	0.65	0.57	0.61	0.63	0.48	0.50	0.44	0.30	0.32	0.33	0.35	0.56	0.53
	Male	Dietary risks	35.33	34.90	34.60	34.41	33.87	33.29	33.18	33.64	33.93	34.84	35.86	35.61	35.38	35.31	33.53	33.73
		High FPG	1.70	2.39	3.34	4.98	7.02	9.12	10.95	12.37	13.20	13.65	13.44	12.93	11.95	10.79	8.93	9.97
		Low phys. act.	4.24	4.59	5.31	5.95	6.68	7.67	8.93	10.37	11.68	12.63	13.47	13.42	13.42	13.44	8.44	9.28
		High BMI	16.08	17.51	18.57	18.91	19.39	19.26	18.66	17.66	16.12	14.51	12.80	12.86	13.04	13.20	17.37	17.00
		Tobacco	4.02	8.90	13.70	17.68	20.50	21.79	22.76	21.97	20.64	18.44	18.07	15.86	13.46	12.09	18.37	18.95
		Alcohol use	2.66	2.96	2.99	2.99	2.91	2.73	2.30	1.71	1.40	0.88	0.61	0.62	0.63	0.62	2.20	1.98
Global	Female	Dietary risks	36.60	35.64	34.79	34.04	33.71	33.42	32.77	32.49	32.53	32.69	33.00	32.65	32.44	32.20	32.90	32.87
		High FPG	1.38	1.94	2.69	3.77	5.08	6.47	7.83	8.89	9.73	10.29	10.37	10.09	9.36	8.37	7.51	7.43
		Low phys. act.	1.97	2.14	2.26	2.50	2.78	3.31	4.04	4.97	6.08	7.22	9.22	9.57	9.83	9.97	5.10	5.06
		High BMI	3.54	4.07	4.32	4.58	4.84	5.15	5.21	5.07	4.73	4.48	3.94	4.03	4.13	4.04	4.62	4.61
		Tobacco	0.68	1.63	2.60	3.60	4.92	6.48	7.14	7.43	6.82	6.65	5.87	5.04	4.42	4.03	5.69	5.64
		Alcohol use	3.56	4.00	4.47	4.71	4.64	5.10	5.00	4.55	4.48	4.06	3.92	4.56	5.23	5.64	4.53	4.52
	Male	Dietary risks	37.93	37.08	36.60	35.88	35.49	34.87	34.18	34.04	34.10	34.18	34.39	33.91	33.76	33.61	34.47	34.41
		High FPG	1.70	2.43	3.34	4.49	5.71	7.15	8.53	9.66	10.66	11.24	11.31	11.03	10.76	10.07	8.08	8.29
		Low phys. act.	1.04	1.14	1.27	1.39	1.61	2.07	2.78	3.62	4.71	5.71	7.40	7.58	7.84	7.82	3.44	3.69
		High BMI	9.57	10.59	11.11	11.34	11.84	12.21	12.23	11.66	10.84	10.10	8.93	9.11	9.96	10.19	11.07	10.95
		Tobacco	3.30	7.43	11.75	15.83	19.33	21.42	22.58	22.81	21.48	19.91	17.98	15.85	12.21	10.38	18.91	18.75
		Alcohol use	13.59	14.25	14.85	15.22	15.38	15.26	14.86	14.13	13.32	12.19	10.91	11.32	11.83	11.75	13.90	13.72

DALYs, disability adjusted life years; NAME, North Africa and Middle East; STD, standardized; FPG, high fasting plasma glucose; BMI, body mass index

**Table 4:** Age trends of percentages of colorectal cancer DALYs attributed to dietary risks factors in NAME and global region in 2019 by gender

Location	Gender	Risk	30-34s	35-39s	40-44s	45-49s	50-54s	55-59s	60-64s	65-69s	70-74s	75-79s	80-84s	85-89s	90-94s	95+	All Ages	Age STD
North Africa and Middle East	Female	Low Whole Grains	20.01	19.87	19.84	19.76	19.75	19.78	19.88	19.94	20.10	20.22	20.48	20.66	20.77	20.85	19.63	19.78
		Low Milk	16.76	16.86	16.71	16.73	16.54	16.24	16.02	15.76	15.47	15.31	14.85	16.30	16.30	16.23	15.85	15.80
		Low Calcium	13.96	13.62	13.19	13.27	12.67	11.83	11.59	11.85	12.07	12.45	13.23	12.81	12.59	12.31	12.31	12.30
		High Red Meat	2.54	2.55	2.51	2.48	2.44	2.41	2.35	2.29	2.21	2.13	1.92	2.06	2.07	2.07	2.30	2.27
		High Processed Meat	1.41	1.40	1.36	1.30	1.27	1.24	1.18	1.13	1.07	1.00	0.87	0.87	0.88	0.88	1.17	1.13
		Low Fiber	1.42	1.32	1.22	1.11	0.89	0.67	0.58	0.64	0.69	0.73	0.85	0.95	0.80	0.81	0.84	0.81
	Male	Low Whole Grains	19.64	19.63	19.61	19.66	19.70	19.81	19.94	20.07	20.29	20.37	20.52	20.62	20.68	20.67	19.62	19.80
		Low Milk	16.90	16.79	16.72	16.60	16.37	16.03	15.77	15.64	15.27	15.50	15.37	16.59	16.55	16.57	15.77	15.74
		Low Calcium	15.27	14.80	14.54	14.42	13.90	13.22	13.11	13.62	13.82	14.96	16.23	15.69	15.38	15.32	13.84	13.96
		High Red Meat	2.60	2.60	2.56	2.51	2.47	2.43	2.37	2.28	2.22	2.12	1.93	2.08	2.09	2.10	2.33	2.29
		High Processed Meat	1.33	1.30	1.26	1.21	1.16	1.12	1.07	1.01	0.94	0.88	0.79	0.79	0.79	0.79	1.07	1.03
		Low Fiber	1.21	1.13	1.07	0.97	0.78	0.62	0.56	0.64	0.70	0.81	1.01	0.98	0.89	0.86	0.78	0.77
Global	Female	Low Whole Grains	15.98	15.74	15.55	15.50	15.41	15.43	15.50	15.54	15.55	15.59	15.72	15.79	15.85	15.72	15.43	15.40
		Low Milk	17.31	17.35	17.15	16.82	16.72	15.97	15.57	15.43	15.20	14.84	14.31	14.53	13.95	13.69	15.48	15.47
		Low Calcium	15.72	14.69	13.78	13.09	12.95	12.43	11.62	11.40	11.48	11.68	12.15	11.15	10.19	9.93	12.04	12.05
		High Red Meat	5.06	5.11	5.20	5.10	5.14	5.21	5.14	4.98	4.83	4.51	3.87	4.39	4.53	4.50	4.81	4.81
		High Processed Meat	2.73	2.88	2.88	2.98	2.95	3.22	3.28	3.25	3.30	3.39	3.39	3.41	3.77	3.88	3.19	3.18
		Low Fiber	2.84	2.60	2.47	2.21	1.95	1.71	1.57	1.58	1.65	1.84	2.31	2.29	2.49	2.52	1.91	1.92
	Male	Low Whole Grains	16.34	16.13	15.99	15.90	15.91	15.86	15.91	15.96	16.06	16.07	16.17	16.10	16.25	16.14	15.85	15.86
		Low Milk	17.48	17.72	17.51	17.18	16.80	16.12	15.71	15.55	15.30	15.05	14.64	14.99	13.96	13.51	15.77	15.68
		Low Calcium	16.92	16.15	15.51	14.83	14.34	13.74	13.03	12.96	13.03	13.28	13.80	12.92	11.75	11.28	13.61	13.55
		High Red Meat	5.95	5.80	5.97	5.87	5.87	5.65	5.46	5.29	5.10	4.78	4.11	4.58	4.63	4.76	5.29	5.23
		High Processed Meat	2.38	2.46	2.52	2.57	2.71	2.95	3.04	3.05	3.13	3.15	3.05	3.00	3.58	3.77	2.91	2.92
		Low Fiber	2.56	2.42	2.33	2.16	1.94	1.72	1.54	1.53	1.56	1.69	2.11	2.06	2.34	2.46	1.80	1.81

DALYs, disability adjusted life years; NAME, North Africa and Middle East; STD, standardized

### National trends of CRC DALYs attributed to lifestyle and metabolic risk factors

Data regarding countries with the lowest and highest CRC DALYs value attributed to each dietary risk factor in 2019 are shown in Table 5. From 1990 to 2019, the CRC DALYs trends of high BMI and HPG were increasing across all NAME countries. The most increase in DALYs rates of high BMI and HPG belonged to Sudan with 217% and Egypt with 197%, and the lowest increase belonged to Bahrain with 9% and UAE with 28%, respectively. Saudi Arabia had the maximum increase in the DALYs rates due to dietary risk (by 35%), smoking (90%) and low physical activity (74%). In contrast, Bahrain had the maximum decrease in DALYs values due to dietary risk (-22%), smoking (-32%) and low physical activity (-8%) during this period **(**Table 5, Supplementary Fig. 3 and Supplementary Fig. 4).

**Table 5:** National trends of colorectal cancer DALYs attributed to risks factors. [DALYs in 1990-DALYS in 2019 (Percentage Change 1990–2019)]

location	Dietary risks	Tobacco	High body-mass index	Low physical activity	High FPG	Alcohol use
Global	107.18–99.79(-7%)	46.45–38.87(-16%)	19.14–24.41(28%)	13.58–12.57(-7%)	18.13–23.33(29%)	32.17–29.07(-10%)
NAME	67.26–72.57(8%)	23.93–25.81(8%)	15.46–27.73(79%)	14.98–20.01(34%)	10.98–21.71(98%)	2.72–2.92(7%)
United Arab Emirates	103.13–94.05(-9%)	33.84–33.65(-1%)	43.93–66.18^*^(51%)	46.99–44.07^*^(-6%)	44.32–56.64(28%)	15.09–6.67(-56%)
Kuwait	46.70-55.65(19%)	18.75–24.77(32%)	20.27–37.78(86%)	18.20-25.02(37%)	16.65–31.36(88%)	0.00-0.28(12,741%)
Qatar	75.85–74.15(-2%)	19.55–23.42(20%)	33.08–54.40(64%)	30.69–35.28(15%)	39.92–66.24^*^(66%)	2.97–2.40(-19%)
Saudi Arabia	48.56–65.58(35%)	8.85–16.80(90%)	13.54–38.11(181%)	17.64–30.68(74%)	12.58–28.82(129%)	1.37–1.09(-20%)
Oman	57.89–55.64(-4%)	13.66–11.36(-17%)	9.32–27.77(198%)	14.93–22.44(50%)	12.09–24.95(106%)	0.73–1.09(49%)
Bahrain	84.29–65.86(-22%)	33.70–23.00(-32%)	32.03–34.93(9%)	26.93–24.74(-8%)	33.88–45.23(33%)	10.86–4.15(-62%)
Turkey	94.81–84.16(-11%)	54.54–46.17(-15%)	29.84–40.82(37%)	21.50-24.28(13%)	17.51–24.16(38%)	7.86–7.69(-2%)
Jordan	113.07-103.39(-9%)	43.32–42.47(-2%)	30.38–46.45(53%)	18.48–22.83(24%)	23.58–32.49(38%)	2.14–2.98(40%)
Libya	95.01-100.48(6%)	29.48–27.85(-6%)	28.80-40.61(41%)	26.10-29.19(12%)	20.40-37.96(86%)	0.34–1.54(346%)
Lebanon	91.41-114.73(26%)	41.79–65.57^*^(57%)	25.15–46.95(87%)	26.32–38.37(46%)	20.94–43.83(109%)	8.61–7.03(-18%)
Tunisia	59.22–62.56(6%)	20.45–24.47(20%)	12.76–23.40(83%)	5.63–8.32^#^(48%)	12.34–25.45(106%)	2.15–4.77(122%)
Iraq	66.08–76.73(16%)	23.58–23.94(2%)	18.86–24.64(31%)	15.56–18.78(21%)	13.37–22.34(67%)	3.43–1.88(-45%)
Iran	65.67–76.29(16%)	16.94–19.53(15%)	10.23–21.82(113%)	12.17–16.20(33%)	8.66–19.85(129%)	0.43–2.07(377%)
Egypt	48.84–55.74(14%)	13.05–22.18(70%)	12.06–26.73(122%)	11.06–16.61(50%)	5.10-15.14(197%)	0.79–1.24(55%)
Algeria	56.49–51.22(-9%)	16.00-16.57(4%)	10.88–19.56(80%)	13.44–16.38(22%)	9.97–19.06(91%)	1.06–2.12(100%)
Syrian Arab Republic	46.54–48.58^#^(4%)	17.63–16.72(-5%)	10.91–15.78(45%)	11.24–13.21(18%)	8.13–14.09(73%)	1.92–1.25(-35%)
Palestine	151.01-187.93^*^(24%)	38.10–50.50(33%)	24.19–42.81(77%)	28.13–39.06(39%)	24.93–56.33(126%)	4.37–8.45^*^(93%)
Morocco	59.83–70.72(18%)	13.23–14.90(13%)	9.60-20.55(114%)	12.28–18.26(49%)	8.06–20.06(149%)	1.81–1.46(-19%)
Sudan	50.90-65.61(29%)	10.34–14.39(39%)	5.24–16.61(217%)	15.43–22.10(43%)	6.63–17.18(159%)	1.45 − 0.08^#^(-94%)
Yemen	57.11–68.96(21%)	15.47–17.99(16%)	3.60–7.82^#^(117%)	9.95–12.38(24%)	5.71–11.32^#^(98%)	2.32–1.21(-48%)
Afghanistan	84.57–89.02(5%)	7.65–11.36^#^(48%)	8.27–13.87(68%)	14.40-16.24(13%)	12.30-23.04(87%)	0.00-0.33(11,636%)

DALYs, disability adjusted life years; NAME, North Africa and Middle East; FPG, high fasting plasma glucose

* Maximum value of attributed DALYs for each risk in 2019

# Minimum value of attributed DALYs for each risk in 2019

### National trends of CRC DALYs attributed to dietary risk factors

Countries with minimum and maximum CRC DALYs attributed to each risk factor in 2019 is shown in Table 6. Between 1990 and 2019, Saudi Arabia had the highest increase in CRC DALYs rates attributable to low intake of whole grain (46%) and low intake of calcium (40%), while Bahrain had the greatest reduction in DALYs values of low intake of whole grain (-20%) and low intake of calcium (-19%). For diet low in calcium, the greatest increase in DALYs rate were seen in UAE (46%) and the greatest decrease was seen in Algeria (-32%). Egypt had the maximum increase in CRC DALYs rate attributable to diet high in red meat (54%) and UAE had the maximum decline (-52%). The highest increase in DALYs value attributed to high processed meat intake belonged to Morocco and Egypt (94%), whereas the highest decrease belonged to UAE (-34%). For diet low in fiber, the greatest increase in DALYs rate was related to Iraq (184%) and the greatest decrease was related to Algeria (-64%) **(**Table 6, Supplementary Fig. 5 and Supplementary Fig. 6).

**Table 6:** National trends of colorectal cancer DALYs attributed to dietary risks factors

location	Low Whole Grains	Low Milk	Low Calcium	High Red Meat	High Processed Meat	Low Fiber
Global	49.42–46.30(-6%)	43.71–46.09(5%)	38.84–38.18(-2%)	15.63–14.95(-4%)	11.71–8.96(-23%)	7.44–5.49(-26%)
NAME	38.49–43.29(12%)	29.57–34.47(17%)	29.47–28.88(-2%)	4.50–4.98(11%)	1.75–2.35(34%)	1.62–1.72(6%)
United Arab Emirates	64.59–57.47(-11%)	63.68–55.51(-13%)	20.36–29.65(46%)	19.79–9.57(-52%)	6.78–4.48^*^(-34%)	2.09–2.45(17%)
Kuwait	27.00-33.73(25%)	29.31–36.52(25%)	13.98–14.99^#^(7%)	6.42–7.74(21%)	1.59–2.23(40%)	2.93–2.52(-14%)
Qatar	50.13–50.46(1%)	45.42–45.52(0%)	20.63–17.22(-17%)	8.10–8.47(5%)	3.11–3.36(8%)	0.78 − 0.69(-11%)
Saudi Arabia	25.58–37.46(46%)	27.40–38.30(40%)	22.79–26.38(16%)	3.38–4.78(41%)	1.33–2.38(78%)	1.13–1.41(24%)
Oman	29.59–32.71(11%)	29.38–32.04(9%)	26.75–18.62(-30%)	4.91–6.64(35%)	1.43–1.89(32%)	2.79–1.39(-50%)
Bahrain	51.21–40.98(-20%)	46.43–37.77(-19%)	31.02–22.33(-28%)	7.26–5.97(-18%)	2.78–2.40(-14%)	1.35–0.90(-34%)
Turkey	66.81–60.97(-9%)	33.56–34.92(4%)	25.24–19.44(-23%)	7.57–6.78(-11%)	2.86–3.25(13%)	0.91 − 0.85(-7%)
Jordan	61.36–60.04(-2%)	52.25–48.33(-7%)	53.03–43.46(-18%)	6.79–6.52(-4%)	2.69–2.85(6%)	6.45–4.75(-26%)
Libya	57.88–59.04(2%)	50.69–52.31(3%)	34.75–40.05(15%)	8.55–7.04(-18%)	3.04–2.99(-2%)	2.14–3.75(76%)
Lebanon	61.76–78.67(27%)	53.67–67.43(26%)	27.09–30.76(14%)	7.90-11.15^*^(41%)	2.86–3.70(29%)	1.03–1.76(71%)
Tunisia	34.04–40.85(20%)	27.95–28.30(1%)	25.82–20.19(-22%)	3.96–4.59(16%)	1.75–2.30(31%)	0.97 − 0.82(-16%)
Iraq	33.06–36.04(9%)	31.88–36.63(15%)	36.66–44.43(21%)	2.94–2.59^#^(-12%)	1.59–1.74(9%)	1.11–3.15(184%)
Iran	34.53–43.54(26%)	29.23–34.52(18%)	33.50-35.14(5%)	4.00-4.44(11%)	1.62–2.15(32%)	2.12–1.41(-34%)
Egypt	21.80-28.52(31%)	26.00-34.34(32%)	28.53–25.81(-10%)	2.96–4.56(54%)	1.36–2.63(94%)	0.48 − 0.47(-3%)
Algeria	31.35–32.91(5%)	26.74–24.34(-9%)	25.19–17.10(-32%)	3.21–3.71(16%)	1.41–1.69(20%)	3.10–1.14(-63%)
Syrian Arab Republic	26.15–28.30^#^(8%)	21.88–22.13^#^(1%)	20.02–19.98(0%)	3.38–3.24(-4%)	1.31–1.34(3%)	1.85–2.09(13%)
Palestine	72.42–88.38^*^(22%)	64.40-80.28^*^(25%)	83.39–105.20^*^(26%)	6.69–7.53(13%)	2.73–3.39(24%)	12.34–16.16^*^(31%)
Morocco	31.34–40.84(30%)	26.64–32.31(21%)	32.79–32.15(-2%)	3.19–4.75(49%)	0.94–1.81(94%)	0.35–0.45^#^(27%)
Sudan	25.00-33.73(35%)	22.34–29.81(33%)	27.54–33.82(23%)	2.50–3.46(38%)	1.00-1.44(45%)	3.33–3.35(0%)
Yemen	27.55–33.09(20%)	25.33–30.03(19%)	32.65–39.24(20%)	2.31–2.86(24%)	1.01–1.21^#^(21%)	3.29–4.62(40%)
Afghanistan	42.51–43.87(3%)	33.06–34.87(5%)	44.39–46.55(5%)	6.17–5.10(-17%)	1.66–1.68(1%)	3.98–7.37(85%)

DALYs, disability adjusted life years; NAME, North Africa and Middle East

* Maximum value of attributed DALYs for each risk in 2019

# Minimum value of attributed DALYs for each risk in 2019

## Discussion

Although the burden of CRC in NAME region was far lower than the world in 2019, the trends of CRC burden and its risk factors were rising faster in NAME in comparison with the world. CRC DALYs increased in all countries with middle and low-middle SDI, whereas some of the high and high-middle SDI countries experienced a decline. Around 60% of CRC burden could be reduced through lifestyle and metabolic factors. Despite the diverse contributions of individual risk factors, almost one-third of CRC DALYs in both females and males were attributable to dietary risk factors in 2019.

A glance at the map indicating CRC DALYs across the globe shows that NAME region had the least burden in comparison with other GBD super regions in 2019. Nevertheless, further detailed evaluation demonstrates that different countries in this region are experiencing diverse levels of burden which entitles specific strategies to address the problem. For example, some countries, such as Lebanon and Palestine with the greatest figures, had a burden like Europe and central Asia, while some others had a smaller burden. In general, the most similar region to NAME in terms of CRC DALYs in 1990 was the region of Latin America and Caribbean. However, in 2019, Latin America and Caribbean experienced greater DALYs than NAME region. This might be due to growing economies, remarkable inequality levels, political instability, and gaps in primary care and health system performance. Indeed, a deeply fragmented health system and poor regulated privatization of public health care in this region cause great challenges in the quality and equity levels of health services, [23–25] whilst people in NAME region (e.g., in Iran) benefit from modern technologies for diagnosis, and extensively available lifesaving treatments as a result of increased hospital bed density and up-to-date facilities [26]. 

Our results are in concordance with the earlier publication which suggested an increasing trend in the incidence of CRC in Asia [5]. In general, comparing countries based on their SDI revealed greater DALYs in countries with high and middle-high SDI in comparison with those in middle- and low-SDI categories. The exceptions for this finding were Kuwait and Oman among high SDI countries and Palestine in low-SDI countries. Unlike the global level [5], most of the high-income countries in NAME region failed to control the burden of CRC. Whereas the lower CRC burden in high SDI countries in the world are attributed to early detection and treatment (due to screening, early referral to physicians and technological developments), the adverse effects of CRC risk factors in high SDI countries in NAME apparently outweigh the beneficial effects of improvements in health systems. For an illustration, the burden of elevated BMI and FPG in high SDI countries in NAME is increasing rapidly, particularly in males, in comparison with the global level and other countries with low and middle SDI in NAME region. Moreover, comparing countries within the region showed that Syrian Arab Republic had the smallest DALYs and showed a slight growth in the DALYs which might be as a result of unchanged dietary habits and reduced alcohol and tobacco use over this time period. In contrast, Palestine and Lebanon with both great DALYs and rise and Saudi Arabia with the greatest rise in CRC DALYs in the region failed to control the burden of all risk factors, except for alcohol in Lebanon and Saudi Arabia.

It seems that unfavorable behavioral changes in lifestyle as a result of industrialization and urbanization are responsible for the rising trend in NAME [27, 28]. We observed that the contributing roles of such unhealthy behaviors increased considerably over this time scale, apart from alcohol which remained almost identical. These changes may exacerbate other metabolic risk factors such as elevated BMI and FPG However, at the global level, CRC DALYs attributable to dietary risks, smoking, low physical activity and alcohol drinking went down from 1990 to 2019, but the corresponding values for elevated BMI and FPG went up. Furthermore, in line with the global results, males in this region had greater rates of DALYs in comparison with females. This might be driven from higher prevalence of visceral adiposity, [29] smoking, [30] and alcohol drinking [31] in males than females. In support of this, comparing risk factors for attributable DALYs between males and females in this region revealed that the shares of elevated BMI, alcohol and tobacco use in CRC DALYs in males are respectively almost 2.5, 4 and 6 times higher than the corresponding values in females, while the contribution of other risk factors is similar. Therefore, beside dietary modifications which are a necessity in both sexes, reducing adiposity, alcohol and tobacco use in males would likely reduce CRC incidence and mortality.

In age group-stratified analysis, the trends of DALYs due to various risk factors, as well as dietary risks, were constantly upward globally in females. However, in males at the global level and both sexes in NAME region, a drop was observed after the age of 85. This decrease might be driven principally by population ageing and a decline in the background mortality rate as a consequence. The constantly growing trend in females at the global level might be caused by cross-influences relating to socio-economic status. On the other hand, in contrast with developed countries, CRC incidence in less-developed countries is mainly observed in individuals younger than 65 and with a greater mortality rate [32]. In addition, both incidence and mortality rates for CRC are higher in males than females [32]. These factors besides greater life expectancy of females compared with males [33] would result in more population ageing and probably a stable growth in the CRC burden in females compared with males across the world. However, lower quality of care for females in comparison with males in some of NAME countries like Afghanistan, Saudi Arabia, and Yemen might cause comparable drops in males and females in this region [34]. 

Low intake of whole grains, milk and calcium were also three main dietary risks which accounted for over 80% of CRC DALYs attributable to dietary risks, with the same order in all age groups. CRC prevention programs need to take some steps to deal with this issue. It is necessary to identify the barriers to consume these food groups. Some of the known barriers include lack of knowledge of the benefits of these food groups and their food sources, family and peers’ effects or social influences, longer preparation time, the economy and income, the low availability and accessibility for instance as a result of exports, or unpleasant taste or other sensory properties for consumers and in contrast wide availability and accessibility to tasty and pleasant alternatives such as refined grains products [35, 36]. After that, the most appropriate actions to deal with the principal barriers should be taken. Governments can take advantages of community-based trials to improve populations’ knowledge (i.e., at schools, public places or on television), controlled exports, subsidize system reinforcement with a focus on whole bread and dairy products, distributing these food groups with lower cost in poor regions, higher tax on refined grain products, and avoiding from unhealthy food advertisement [37, 38]. These behavioral modifications consequently can improve metabolic risk factors, such as elevated BMI and FPG.

All GBD limitations also come with the present analysis which need to be taken into consideration. First, the diversity in the measurement tools and definition of risk factors, for example low whole grains consumption, may impact types of errors and consequently the accuracy of results. Detection biases and changes in screening protocols over time are other examples. Second, the collinearity between various risk factors or healthy behaviors with each other may cause an overestimation of the real effect sizes. Third, in prospective observational studies, it is not possible to determine when the share of an individual dietary component in total energy intake goes up, which food group has been restricted. In other words, it is not viable to illustrate the absolute level of a dietary component and just the associations are reported for their contribution to daily energy intake. Fourth, due to the lack of data on cancer estimates in some countries, these estimates come either from predictive covariates or from figures in neighboring countries. This is also true for the estimates for the early years of GBD (30 years ago) and the most recent years, which rely on preceding trends and covariates owing to the non-availability data. Finally, our estimations are based on some observational studies, not clinical trials, and therefore cannot reveal any causality. This study has also its own strengths. It is the first comprehensive review in NAME region which explored CRC and its risk factors burden at national level and shed light on required strategies to prevent CRC in each country in the region.

In summary, we found that the burdens of CRC and its risk factors in NAME region are rising faster than the world, particularly in males. Except for Palestine, as a low SDI country with the highest CRC DALYs in the region, the burden of CRC was higher in countries with high and middle-high SDI in comparison with the low and middle SDI countries. Dietary risk factors were the leading contributors to CRC DALYs. The highest proportion of DALYs attributed to diet was due to low whole grains consumption. Taking actions to minimize the prevalence of modifiable risk factors, in particular in high SDI countries, and improving screening are crucial to reduce CRC incidence and burden.

### Electronic supplementary material

Below is the link to the electronic supplementary material.

Supplementary Material 1

Supplementary Material 2

## Data Availability

Publicly available datasets were analyzed in current study. The data can be found here: http://ghdx.healthdata.org/gbd-results-tool.
