# Correction to: Burden of colorectal cancer and its risk factors in the North Africa and Middle East (NAME) region, 1990–2019: a systematic analysis of the global burden of disease study

**DOI:** 10.1186/s12889-024-18666-9

**Published:** 2024-05-15

**Authors:** Fahimeh Haghighatdoost, Kamran Mehrabani-Zeinabad, Parisa Hajihashemi, Noushin Mohammadifard, Peyman Adibi

**Affiliations:** 1 Isfahan Cardiovascular Research Center, Cardiovascular Research Institute, Isfahan University of Medical Sciences, Isfahan, Iran; 2 Isfahan Gastroenterology and Hepatology Research Center, Isfahan University of Medical Sciences, Isfahan, Iran

**Correction to: BMC Public Health 24, 557 (2024)**

10.1186/s12889-024-18027-6

The original publication of this article contained 1 error related to the author information:

1. The correct contribution statement is “**Fahimeh Haghighatdoost and Kamran Mehrabani-Zeinabad contributed equally and both are first authors”**.

The original article has been updated to correct these errors.

