# Supplementary material for: Burden of colorectal cancer and its risk factors in the North Africa and Middle East (NAME) region, 1990–2019: a systematic analysis of the global burden of disease study
Source: BMC Public Health. 2024 Feb 22;24:557. doi: 10.1186/s12889-024-18027-6 (PMC10882825; doi:10.1186/s12889-024-18027-6)
Supplement: Supplementary file 1 — Supplementary Material 1 [file 12889_2024_18027_MOESM1_ESM.docx]

**Supplementary Table 1.**

|  |  | Causes of death in different levels of classifications in GBD. |
| --- | --- | --- |
| Level 1 | All causes | Communicable, mental, neonatal and nutritional diseases |
|  |  | Injuries |
|  |  | **Non-communicable diseases** |
| Level 2 | Communicable, mental, neonatal and nutritional diseases | Enteric infections, HIV/AIDS and sexually transmitted infections, Maternal and neonatal disorders, Neglected tropical diseases and malaria, Nutritional deficiencies, Other infectious diseases, Respiratory infections and tuberculosis |
|  | Injuries | Self-harm and interpersonal violence, Transport injuries, Unintentional injuries |
|  | Non-communicable diseases | Cardiovascular diseases, Chronic respiratory diseases, Diabetes and kidney diseases, Digestive diseases, Mental disorders, Musculoskeletal disorders, **Neoplasms**, Neurological disorders, Other non-communicable diseases, Sense organ diseases, Skin and subcutaneous diseases, Substance use disorders |
| Level 3 | Neoplasms | Bladder cancer, Brain and central nervous system cancer, Breast cancer, Cervical cancer, **Colon and rectum cancer**, Esophageal cancer, Gallbladder and biliary tract cancer, Hodgkin lymphoma, Kidney cancer, Larynx cancer, Leukemia, Lip and oral cavity cancer, Liver cancer, Malignant skin melanoma, Mesothelioma, Multiple myeloma, Nasopharynx cancer, Non-Hodgkin lymphoma, Non-melanoma skin cancer  Other malignant neoplasms, Other neoplasms, Other pharynx cancer, Ovarian cancer, Pancreatic cancer, Prostate cancer, Stomach cancer, Testicular cancer, Thyroid cancer, Tracheal, bronchus, and lung cancer, Uterine cancer |

**Supplementary Table 2 - Trends of deaths, DALYs, YLL, YLD, prevalence and incidence rates of** **colorectal cancer in NAME and global region**

| Measure | Location | Gender | Year | | | | | Percentage Change  1990-2019 |
| --- | --- | --- | --- | --- | --- | --- | --- | --- |
|  |  |  | 1990 | 2000 | 2010 | 2015 | 2019 |  |
| Deaths | Global | Female | 12.91  (12.03,13.64) | 12.67  (11.70,13.26) | 11.72  (10.71,12.35) | 11.20  (10.08,11.86) | 11.24  (10.01,12.17) | -12.97% |
|  |  | Male | 16.16  (15.44,16.93) | 16.54  (15.84,17.06) | 17.02  (16.16,17.72) | 16.53  (15.58,17.40) | 16.64  (15.39,17.85) | 2.97% |
|  | NAME | Female | 7.94  (6.69,9.38) | 7.59  (6.88,8.29) | 8.82  (8.02,9.51) | 9.11  (8.27,9.95) | 9.18  (8.04,10.43) | 15.60% |
|  |  | Male | 8.42  (6.78,10.21) | 7.95  (7.21,8.78) | 9.57  (8.83,10.33) | 10.26  (9.38,11.23) | 10.45  (9.17,11.90) | 24.09% |
| DALYs | Global | Female | 276.80  (261.99,293.17) | 267.57  (254.53,279.64) | 246.54  (232.85,257.87) | 236.92  (221.45,249.04) | 237.93  (218.73,257.07) | -14.04% |
|  |  | Male | 346.63  (330.92,366.69) | 353.88  (342.42,364.64) | 368.23  (353.02,383.47) | 357.58  (338.78,377.03) | 360.05  (333.07,387.83) | 3.87% |
|  | NAME | Female | 185.93  (158.40,223.33) | 175.72  (161.94,191.48) | 194.61  (179.43,209.80) | 199.37  (182.86,218.46) | 200.40  (175.46,229.48) | 7.78% |
|  |  | Male | 199.28  (161.64,244.27) | 186.07  (169.90,206.31) | 216.69  (201.14,234.57) | 231.38  (212.58,253.38) | 236.23  (206.22,269.49) | 18.54% |
| YLLs | Global | Female | 267.69  (253.11,284.25) | 257.85  (245.65,269.82) | 236.50  (223.33,247.93) | 227.02  (212.34,238.91) | 227.74  (209.51,246.84) | -14.92% |
|  |  | Male | 335.02  (319.69,354.41) | 340.81  (330.00,351.35) | 352.86  (338.30,367.60) | 341.92  (324.19,359.91) | 343.77  (317.10,371.07) | 2.61% |
|  | NAME | Female | 182.84  (155.75,220.23) | 172.55  (158.87,188.26) | 190.70  (175.52,205.74) | 195.13  (178.63,213.54) | 195.91  (171.44,224.08) | 7.14% |
|  |  | Male | 195.47  (158.40,240.15) | 181.99  (166.35,202.18) | 210.93  (195.51,228.46) | 224.75  (206.27,246.65) | 228.95  (200.00,261.03) | 17.13% |
| YLDs | Global | Female | 9.11  (6.75,11.67) | 9.71  (7.23,12.44) | 10.04  (7.41,13.02) | 9.90  (7.31,12.78) | 10.19  (7.44,13.26) | 11.85% |
|  |  | Male | 11.62  (8.64,14.99) | 13.06  (9.69,16.87) | 15.37  (11.49,19.85) | 15.65  (11.44,20.27) | 16.28  (11.96,21.13) | 40.19% |
|  | NAME | Female | 3.09  (2.15,4.15) | 3.17  (2.29,4.12) | 3.91  (2.84,5.07) | 4.24  (3.10,5.57) | 4.50  (3.28,5.95) | 45.65% |
|  |  | Male | 3.81  (2.54,5.28) | 4.09  (2.98,5.31) | 5.76  (4.26,7.41) | 6.63  (4.83,8.62) | 7.29  (5.32,9.67) | 91.17% |
| Prevalence | Global | Female | 92.63  (88.57,96.74) | 100.65  (96.07,103.77) | 107.60  (102.04,111.78) | 106.95  (100.60,112.15) | 110.88  (100.46,120.99) | 19.71% |
|  |  | Male | 112.66  (108.56,117.54) | 129.89  (126.50,133.44) | 158.72  (152.52,164.99) | 163.44  (155.36,171.65) | 171.27  (156.49,188.19) | 52.03% |
|  | NAME | Female | 25.42  (21.88,30.24) | 27.06  (24.99,29.29) | 35.01  (32.41,37.53) | 38.84  (35.89,42.06) | 42.17  (37.13,47.60) | 65.90% |
|  |  | Male | 32.43  (26.60,38.94) | 36.68  (33.74,40.16) | 55.17  (51.19,59.28) | 65.27  (60.06,71.57) | 73.25  (64.23,83.77) | 125.84% |
| Incidence | Global | Female | 19.92  (18.83,20.81) | 21.10  (19.82,21.90) | 21.20  (19.64,22.20) | 20.72  (19.18,21.84) | 21.22  (19.05,23.16) | 6.51% |
|  |  | Male | 25.19  (24.24,26.11) | 28.02  (27.08,28.79) | 31.85  (30.34,33.25) | 32.02  (30.21,33.66) | 33.06  (30.22,36.15) | 31.23% |
|  | NAME | Female | 8.35  (7.08,9.89) | 8.37  (7.68,9.07) | 10.26  (9.39,11.04) | 10.96  (10.03,11.96) | 11.43  (10.04,12.96) | 36.91% |
|  |  | Male | 9.64  (7.80,11.75) | 9.91  (9.04,10.90) | 13.44  (12.46,14.49) | 15.19  (13.95,16.68) | 16.31  (14.29,18.65) | 69.14% |

DALYs, disability adjusted life years; YLL, years of life lost; YLD, years lived with disability; NAME, North Africa and Middle East.

**Supplementary Table 3– colorectal cancer DALYs in the GBD super regions and NAME countries in 2019.**

| **SDI/**  **Region** | **Location** | **1990** | **2019** | **Percentage Change**  **1990-2019** |
| --- | --- | --- | --- | --- |
| - | Global | 308.51  (294.71,320.75) | 295.54  (275.17,312.98) | -4.20% |
| Super Regions | North Africa and Middle East | 192.83  (162.72,227.03) | 218.67  (194.05,246.53) | 13.40% |
|  | Central Europe, Eastern Europe,  and Central Asia | 440.13  (427.91,451.24) | 433.22  (399.15,469.87) | -1.57% |
|  | High-income | 445.81  (432.26,455.61) | 347.90  (329.30,360.47) | -21.96% |
|  | Latin America and Caribbean | 204.56  (197.02,210.18) | 251.70  (231.16,275.37) | 23.05% |
|  | South Asia | 125.38  (111.12,142.89) | 165.06  (141.74,189.86) | 31.65% |
|  | Southeast Asia, East Asia,  and Oceania | 245.08  (220.24,269.88) | 326.46  (287.75,365.26) | 33.21% |
|  | Sub-Saharan Africa | 166.59  (140.44,192.12) | 191.02  (168.15,214.97) | 14.66% |
| High  SDI | United Arab Emirates | 362.51  (235.62,488.39) | 327.12  (223.85,452.27) | -9.76% |
|  | Kuwait | 160.01  (142.70,177.66) | 202.28  (168.79,242.15) | 26.42% |
|  | Qatar | 271.89  (204.05,341.65) | 282.97  (218.24,354.53) | 4.08% |
| High-middle  SDI | Saudi Arabia | 151.74  (102.99,211.15) | 223.14  (175.67,276.01) | 47.05% |
|  | Oman | 170.59  (116.59,233.90) | 199.12  (162.35,243.38) | 16.72% |
|  | Bahrain | 268.98  (223.12,316.41) | 223.72  (174.97,280.03) | -16.83% |
|  | Turkey | 318.96  (256.03,391.02) | 289.17  (230.39,354.79) | -9.34% |
|  | Jordan | 289.80  (236.02,354.12) | 282.69  (235.46,339.66) | -2.45% |
|  | Libya | 286.86  (203.87,417.79) | 298.15  (217.65,385.98) | 3.93% |
|  | Lebanon | 293.08  (228.23,371.22) | 372.53  (300.31,471.78) | 27.11% |
| Middle  SDI | Tunisia | 172.74  (140.32,205.78) | 208.08  (151.00,280.73) | 20.46% |
|  | Iraq | 171.88  (125.56,238.92) | 195.18  (151.48,247.35) | 13.56% |
|  | Iran | 165.60  (136.58,198.69) | 206.73  (192.19,222.07) | 24.84% |
|  | Egypt | 138.04  (126.92,150.01) | 184.40  (133.58,253.62) | 33.58% |
|  | Algeria | 156.73  (128.28,189.89) | 166.05  (130.81,206.55) | 5.94% |
|  | Syrian Arab Republic | 135.34  (104.68,172.59) | 140.02  (102.06,185.69) | 3.46% |
| Low-middle  SDI | Palestine | 348.53  (234.65,485.15) | 434.66  (368.82,503.88) | 24.71% |
|  | Morocco | 147.88  (119.17,173.31) | 193.64  (145.41,245.01) | 30.94% |
|  | Sudan | 121.33  (91.37,179.24) | 164.39  (116.89,242.57) | 35.49% |
| Low  SDI | Yemen | 130.88  (84.43,191.18) | 156.04  (116.18,211.94) | 19.22% |
|  | Afghanistan | 202.57  (115.72,317.70) | 209.28  (137.22,281.82) | 3.32% |

DALYs, disability adjusted life years; GBD, global burden of diseases; SDI, Socio-demographic Index; NAME, North Africa and Middle East.

**Supplementary Table 4 - Age trends of colorectal cancer DALYs attributed to risks in NAME and global region in 2019 by gender.**

| **Location** | **Gender** | **Risk** | **30-34s** | **35-39s** | **40-44s** | **45-49s** | **50-54s** | **55-59s** | **60-64s** | **65-69s** | **70-74s** | **75-79s** | **80-84s** | **85-89s** | **90-94s** | **95+** |
| --- | --- | --- | --- | --- | --- | --- | --- | --- | --- | --- | --- | --- | --- | --- | --- | --- |
| North Africa and Middle East | Female | Dietary risks | 21.32 | 33.57 | 53.20 | 89.24 | 131.23 | 165.17 | 208.68 | 254.75 | 327.13 | 393.78 | 472.95 | 498.19 | 402.98 | 342.31 |
|  |  | High FPG | 1.05 | 2.39 | 5.40 | 13.57 | 28.14 | 46.53 | 71.92 | 98.29 | 133.85 | 164.04 | 189.80 | 191.59 | 142.26 | 104.68 |
|  |  | Low phys. act. | 3.23 | 5.27 | 8.99 | 16.44 | 26.62 | 37.94 | 56.60 | 79.30 | 112.29 | 144.01 | 178.38 | 187.40 | 151.39 | 129.42 |
|  |  | High BMI | 3.68 | 6.55 | 11.57 | 20.51 | 32.60 | 42.45 | 53.35 | 61.91 | 74.12 | 80.80 | 83.97 | 89.22 | 73.23 | 62.72 |
|  |  | Tobacco | 0.26 | 0.88 | 2.64 | 5.47 | 12.26 | 20.13 | 26.73 | 33.73 | 37.95 | 35.26 | 37.31 | 29.01 | 21.99 | 17.62 |
|  |  | Alcohol use | 0.40 | 0.59 | 1.15 | 1.71 | 2.27 | 3.10 | 4.08 | 3.81 | 5.08 | 5.26 | 4.20 | 4.71 | 4.01 | 3.61 |
|  |  | All risk factors | 26.74 | 43.42 | 71.77 | 124.89 | 193.72 | 256.85 | 337.37 | 420.26 | 542.72 | 648.30 | 763.83 | 795.58 | 637.15 | 533.36 |
|  | Male | Dietary risks | 23.04 | 36.28 | 57.72 | 93.34 | 145.04 | 211.46 | 300.84 | 363.91 | 447.17 | 478.27 | 487.15 | 488.37 | 429.95 | 386.31 |
|  |  | High FPG | 1.11 | 2.49 | 5.59 | 13.56 | 30.09 | 57.97 | 99.36 | 134.19 | 173.97 | 187.58 | 182.77 | 177.49 | 145.49 | 118.23 |
|  |  | Low phys. act. | 2.75 | 4.75 | 8.83 | 16.08 | 28.46 | 48.63 | 80.73 | 111.85 | 153.93 | 173.35 | 182.95 | 183.91 | 162.95 | 146.92 |
|  |  | High BMI | 10.49 | 18.20 | 30.96 | 51.30 | 83.04 | 122.38 | 169.16 | 191.01 | 212.45 | 199.23 | 174.07 | 176.37 | 158.69 | 144.71 |
|  |  | Tobacco | 2.62 | 9.26 | 22.85 | 47.98 | 87.81 | 138.44 | 206.34 | 237.52 | 272.16 | 253.16 | 245.46 | 217.56 | 163.59 | 132.36 |
|  |  | Alcohol use | 1.73 | 3.07 | 4.99 | 8.11 | 12.45 | 17.32 | 20.89 | 18.52 | 18.44 | 12.16 | 8.27 | 8.56 | 7.67 | 6.78 |
|  |  | All risk factors | 34.20 | 58.17 | 99.02 | 168.74 | 275.97 | 417.66 | 606.18 | 726.91 | 881.54 | 907.75 | 896.72 | 887.56 | 768.70 | 680.63 |
| Global | Female | Dietary risks | 24.26 | 37.89 | 60.89 | 94.92 | 144.53 | 194.86 | 255.31 | 315.37 | 400.75 | 486.73 | 572.63 | 628.79 | 687.82 | 758.48 |
|  |  | High FPG | 0.91 | 2.07 | 4.72 | 10.52 | 21.80 | 37.69 | 61.02 | 86.27 | 119.83 | 153.20 | 180.02 | 194.46 | 198.50 | 197.45 |
|  |  | Low phys. act. | 1.31 | 2.27 | 3.95 | 6.96 | 11.87 | 19.29 | 31.41 | 48.14 | 74.82 | 107.37 | 160.00 | 184.48 | 208.64 | 235.03 |
|  |  | High BMI | 2.35 | 4.33 | 7.58 | 12.79 | 20.76 | 30.04 | 40.64 | 49.19 | 58.31 | 66.76 | 68.43 | 77.58 | 87.42 | 94.97 |
|  |  | Tobacco | 0.45 | 1.73 | 4.54 | 10.03 | 21.05 | 37.74 | 55.64 | 72.08 | 83.95 | 98.98 | 101.95 | 97.16 | 93.72 | 94.92 |
|  |  | Alcohol use | 2.35 | 4.25 | 7.81 | 13.12 | 19.88 | 29.69 | 38.95 | 44.17 | 55.11 | 60.50 | 68.06 | 87.90 | 110.89 | 132.79 |
|  |  | All risk factors | 28.75 | 46.78 | 78.21 | 127.17 | 201.81 | 286.70 | 390.64 | 493.20 | 632.72 | 774.12 | 913.08 | 1008.40 | 1103.13 | 1210.34 |
|  | Male | Dietary risks | 37.70 | 57.74 | 89.35 | 140.73 | 216.42 | 313.81 | 432.60 | 548.69 | 671.11 | 774.14 | 860.65 | 953.80 | 878.52 | 824.84 |
|  |  | High FPG | 1.69 | 3.80 | 8.17 | 17.62 | 34.84 | 64.37 | 107.97 | 155.60 | 209.73 | 254.57 | 283.00 | 310.21 | 279.93 | 247.24 |
|  |  | Low phys. act. | 1.03 | 1.77 | 3.09 | 5.41 | 9.78 | 18.55 | 34.98 | 58.13 | 92.45 | 129.20 | 185.09 | 213.22 | 204.11 | 191.75 |
|  |  | High BMI | 9.51 | 16.47 | 27.10 | 44.42 | 72.08 | 109.77 | 154.60 | 187.76 | 213.18 | 228.49 | 223.51 | 256.15 | 259.40 | 250.03 |
|  |  | Tobacco | 3.28 | 11.57 | 28.68 | 62.13 | 117.88 | 192.76 | 285.53 | 367.39 | 422.45 | 450.61 | 449.88 | 445.73 | 317.88 | 254.71 |
|  |  | Alcohol use | 13.50 | 22.18 | 36.24 | 59.67 | 93.73 | 137.29 | 188.01 | 227.59 | 261.96 | 275.95 | 272.93 | 318.38 | 307.82 | 288.27 |
|  |  | All risk factors | 54.07 | 88.66 | 145.47 | 242.28 | 390.16 | 588.34 | 836.93 | 1069.86 | 1298.81 | 1477.51 | 1607.05 | 1777.93 | 1615.50 | 1495.52 |

DALYs, disability adjusted life years; NAME, North Africa and Middle East; FPG, high fasting plasma glucose; BMI**,** body mass index.

**Supplementary Table 5 - Age trends of colorectal cancer DALYs attributed to dietary risks factors in NAME and global region in 2019 by gender.**

| **Location** | **Gender** | **Risk** | **30-34s** | **35-39s** | **40-44s** | **45-49s** | **50-54s** | **55-59s** | **60-64s** | **65-69s** | **70-74s** | **75-79s** | **80-84s** | **85-89s** | **90-94s** | **95+** |
| --- | --- | --- | --- | --- | --- | --- | --- | --- | --- | --- | --- | --- | --- | --- | --- | --- |
| North Africa and Middle East | Female | Low Whole Grains | 12.27 | 19.44 | 31.19 | 52.31 | 78.43 | 101.33 | 129.55 | 157.63 | 202.52 | 242.81 | 289.42 | 307.51 | 251.57 | 215.35 |
|  |  | Low Milk | 10.29 | 16.49 | 26.26 | 44.30 | 65.66 | 83.12 | 104.44 | 124.47 | 155.65 | 183.72 | 209.71 | 242.34 | 196.79 | 167.40 |
|  |  | Low Calcium | 8.56 | 13.31 | 20.71 | 35.10 | 50.27 | 60.49 | 75.42 | 93.51 | 121.39 | 149.29 | 186.74 | 190.52 | 152.10 | 126.82 |
|  |  | High Red Meat | 1.56 | 2.49 | 3.94 | 6.54 | 9.63 | 12.28 | 15.31 | 18.04 | 22.20 | 25.51 | 27.06 | 30.59 | 24.96 | 21.35 |
|  |  | High Processed Meat | 0.87 | 1.37 | 2.14 | 3.45 | 5.05 | 6.35 | 7.71 | 8.93 | 10.76 | 11.99 | 12.30 | 13.05 | 10.63 | 9.11 |
|  |  | Low Fiber | 0.87 | 1.29 | 1.92 | 2.93 | 3.52 | 3.40 | 3.75 | 5.02 | 6.88 | 8.71 | 12.03 | 14.18 | 9.66 | 8.32 |
|  | Male | Low Whole Grains | 12.81 | 20.43 | 32.73 | 53.34 | 84.40 | 125.87 | 180.82 | 217.20 | 267.56 | 279.80 | 278.82 | 282.90 | 251.40 | 226.22 |
|  |  | Low Milk | 11.03 | 17.46 | 27.90 | 45.03 | 70.18 | 101.82 | 142.94 | 169.04 | 201.14 | 212.68 | 208.40 | 227.35 | 200.90 | 181.02 |
|  |  | Low Calcium | 9.96 | 15.37 | 24.25 | 39.11 | 59.51 | 83.97 | 118.75 | 147.24 | 181.97 | 205.30 | 220.43 | 215.05 | 186.94 | 167.61 |
|  |  | High Red Meat | 1.70 | 2.69 | 4.27 | 6.81 | 10.56 | 15.45 | 21.42 | 24.67 | 29.19 | 29.14 | 26.18 | 28.46 | 25.24 | 22.90 |
|  |  | High Processed Meat | 0.87 | 1.35 | 2.11 | 3.27 | 4.99 | 7.14 | 9.67 | 10.92 | 12.47 | 12.08 | 10.70 | 10.90 | 9.64 | 8.64 |
|  |  | Low Fiber | 0.79 | 1.18 | 1.79 | 2.64 | 3.33 | 3.93 | 5.09 | 6.88 | 9.18 | 11.15 | 13.67 | 13.38 | 10.83 | 9.34 |
| Global | Female | Low Whole Grains | 10.59 | 16.73 | 27.23 | 43.21 | 66.07 | 89.96 | 120.76 | 150.87 | 191.64 | 232.19 | 272.84 | 303.98 | 335.95 | 370.14 |
|  |  | Low Milk | 11.47 | 18.45 | 30.03 | 46.92 | 71.75 | 93.16 | 121.32 | 149.77 | 187.24 | 220.91 | 248.09 | 279.62 | 295.42 | 322.04 |
|  |  | Low Calcium | 10.42 | 15.62 | 24.12 | 36.51 | 55.55 | 72.50 | 90.61 | 110.70 | 141.51 | 174.00 | 210.98 | 214.79 | 216.27 | 234.25 |
|  |  | High Red Meat | 3.35 | 5.43 | 9.09 | 14.23 | 22.01 | 30.36 | 40.03 | 48.36 | 59.40 | 67.08 | 66.99 | 84.39 | 95.87 | 105.66 |
|  |  | High Processed Meat | 1.81 | 3.06 | 5.04 | 8.29 | 12.65 | 18.75 | 25.55 | 31.56 | 40.66 | 50.54 | 58.85 | 65.78 | 79.99 | 91.51 |
|  |  | Low Fiber | 1.88 | 2.76 | 4.32 | 6.15 | 8.36 | 9.98 | 12.27 | 15.31 | 20.27 | 27.34 | 39.99 | 44.10 | 52.78 | 59.33 |
|  | Male | Low Whole Grains | 16.24 | 25.12 | 39.05 | 62.39 | 97.06 | 142.78 | 201.40 | 257.37 | 316.06 | 363.98 | 404.75 | 452.93 | 422.83 | 395.97 |
|  |  | Low Milk | 17.36 | 27.59 | 42.74 | 67.39 | 102.47 | 145.09 | 198.81 | 250.62 | 301.10 | 340.79 | 366.36 | 421.24 | 362.90 | 331.10 |
|  |  | Low Calcium | 16.81 | 25.16 | 37.86 | 58.19 | 87.50 | 123.64 | 164.94 | 208.98 | 256.40 | 300.83 | 345.58 | 363.50 | 306.07 | 277.10 |
|  |  | High Red Meat | 5.92 | 9.03 | 14.58 | 23.02 | 35.78 | 50.86 | 69.09 | 85.23 | 100.34 | 108.34 | 102.67 | 128.55 | 120.24 | 116.66 |
|  |  | High Processed Meat | 2.36 | 3.83 | 6.14 | 10.05 | 16.49 | 26.54 | 38.44 | 49.14 | 61.53 | 71.29 | 76.31 | 84.28 | 93.25 | 92.55 |
|  |  | Low Fiber | 2.55 | 3.76 | 5.68 | 8.48 | 11.84 | 15.47 | 19.53 | 24.68 | 30.73 | 38.30 | 52.82 | 57.89 | 60.78 | 60.35 |

DALYs, disability adjusted life years; NAME, North Africa and Middle East.
