# Supplementary material for: Burden of colorectal cancer and its risk factors in the North Africa and Middle East (NAME) region, 1990–2019: a systematic analysis of the global burden of disease study
Source: BMC Public Health. 2024 Feb 22;24:557. doi: 10.1186/s12889-024-18027-6 (PMC10882825; doi:10.1186/s12889-024-18027-6)
Supplement: Supplementary file 2 — Supplementary Material 2 [file 12889_2024_18027_MOESM2_ESM.docx]

**
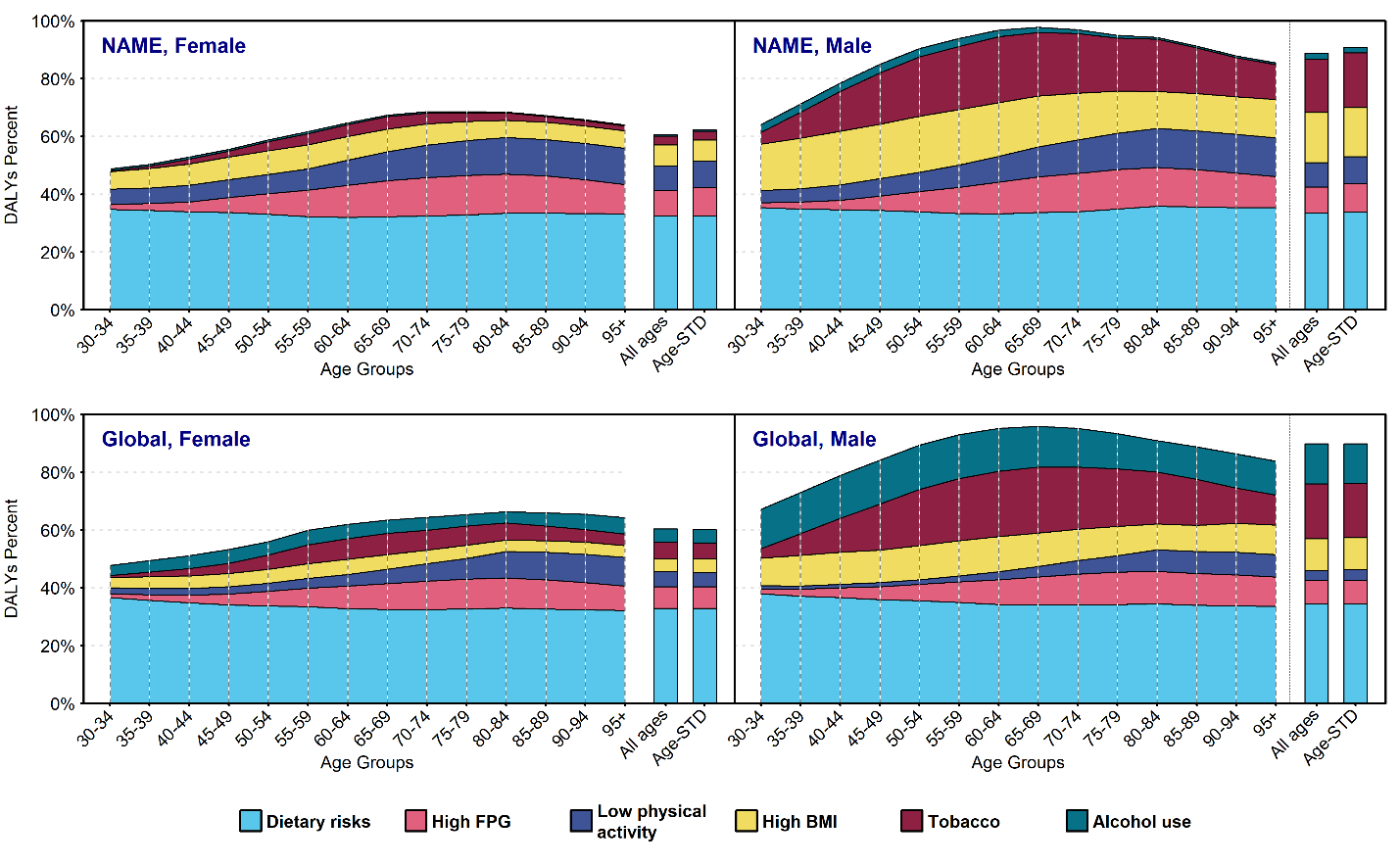
**

**Supplementary Figure 1.** Age trends of risks attributed to colorectal cancer DALYs in NAME and global region in 2019 by gender. DALYs, disability adjusted life years; NAME, North Africa and Middle East; FPG, high fasting plasma glucose.

**
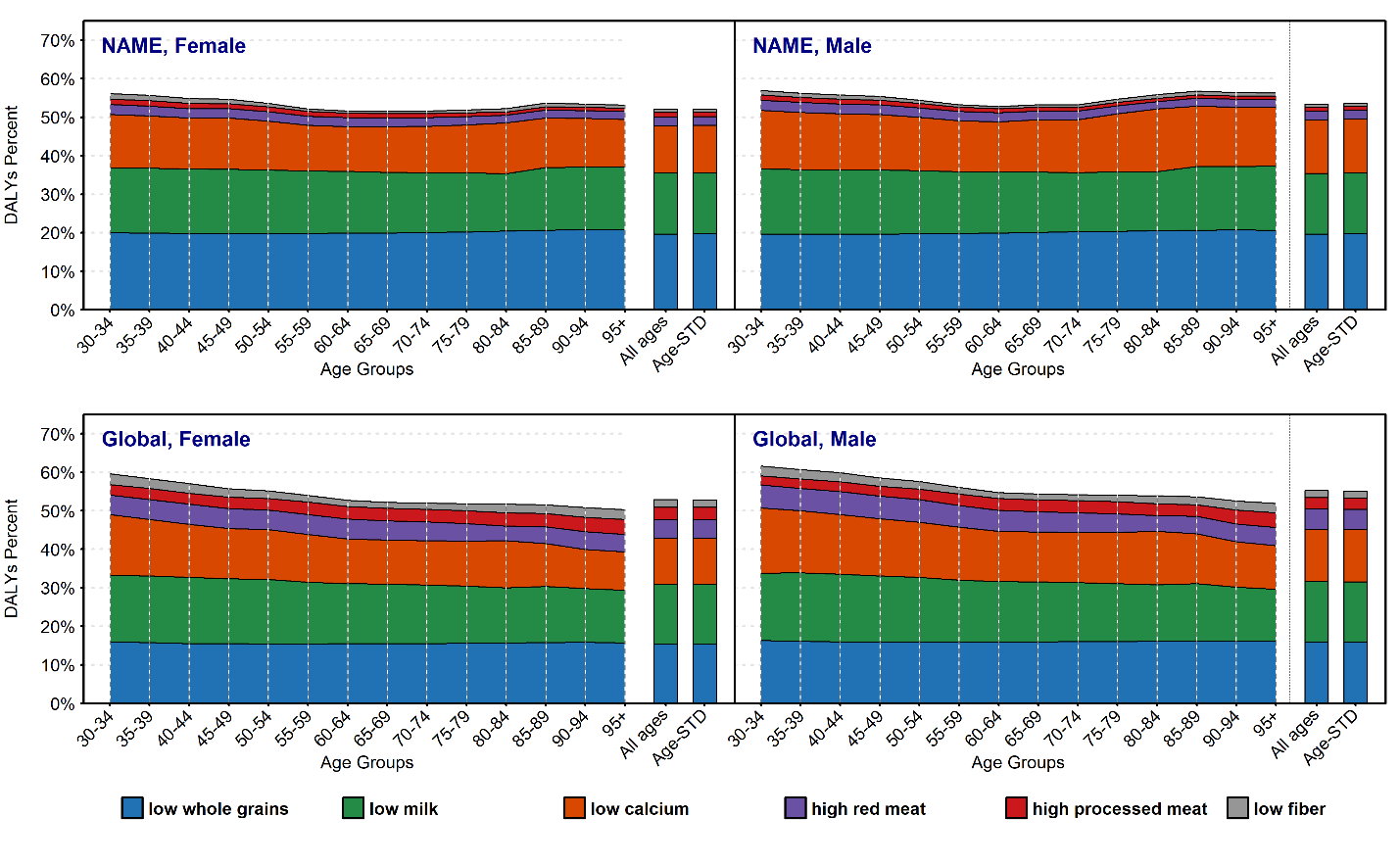
**

**Supplementary Figure 2.** Age trends of colorectal cancer DALYs attributed to dietary risks factors in NAME and global region in 2019 by gender. DALYs, disability adjusted life years; NAME, North Africa and Middle East.

**
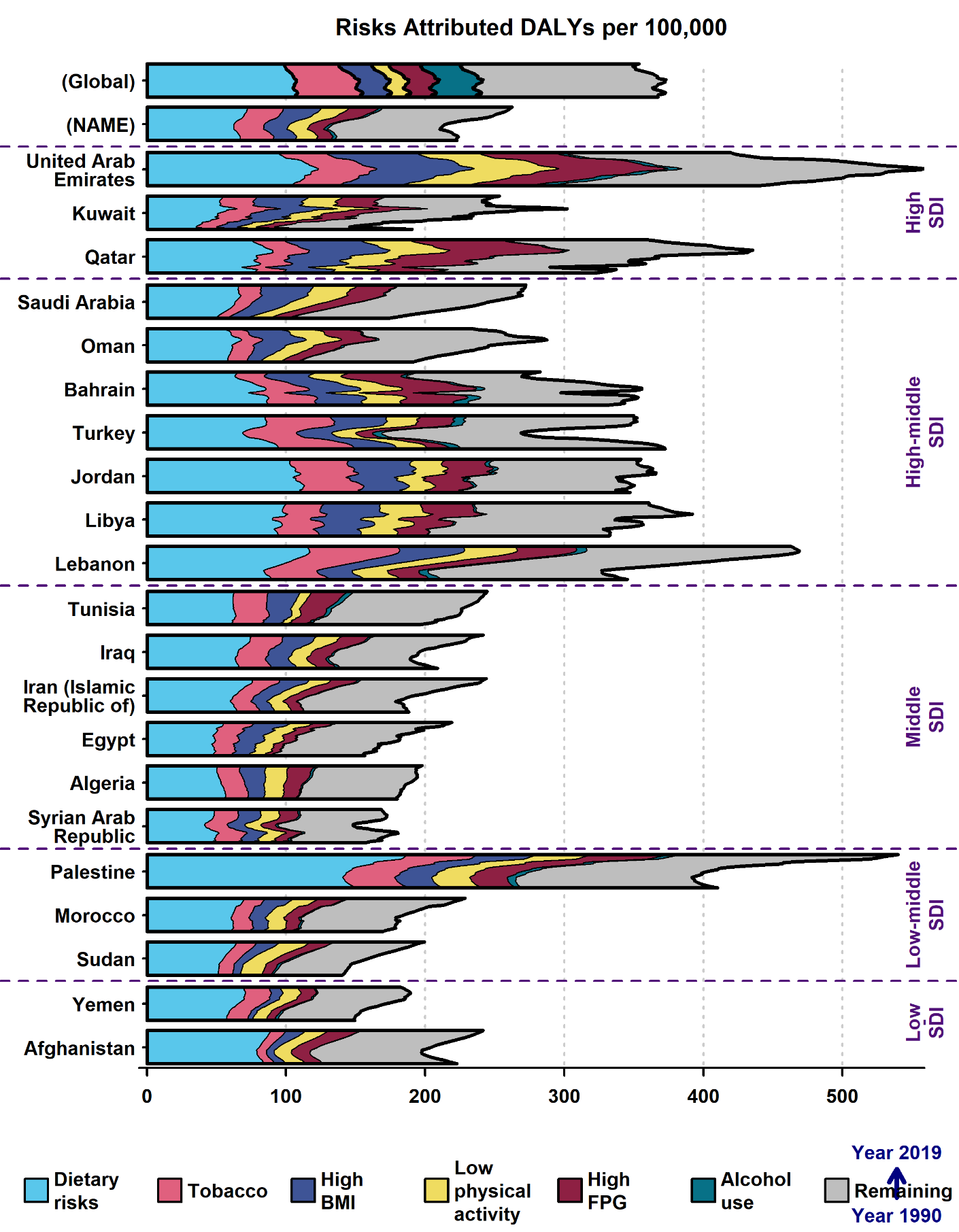
**

**Supplementary Figure 3.** National trends of colorectal cancer DALYs attributed to risks factors by gender. DALYs, disability adjusted life years; FPG, high fasting plasma glucose.

**
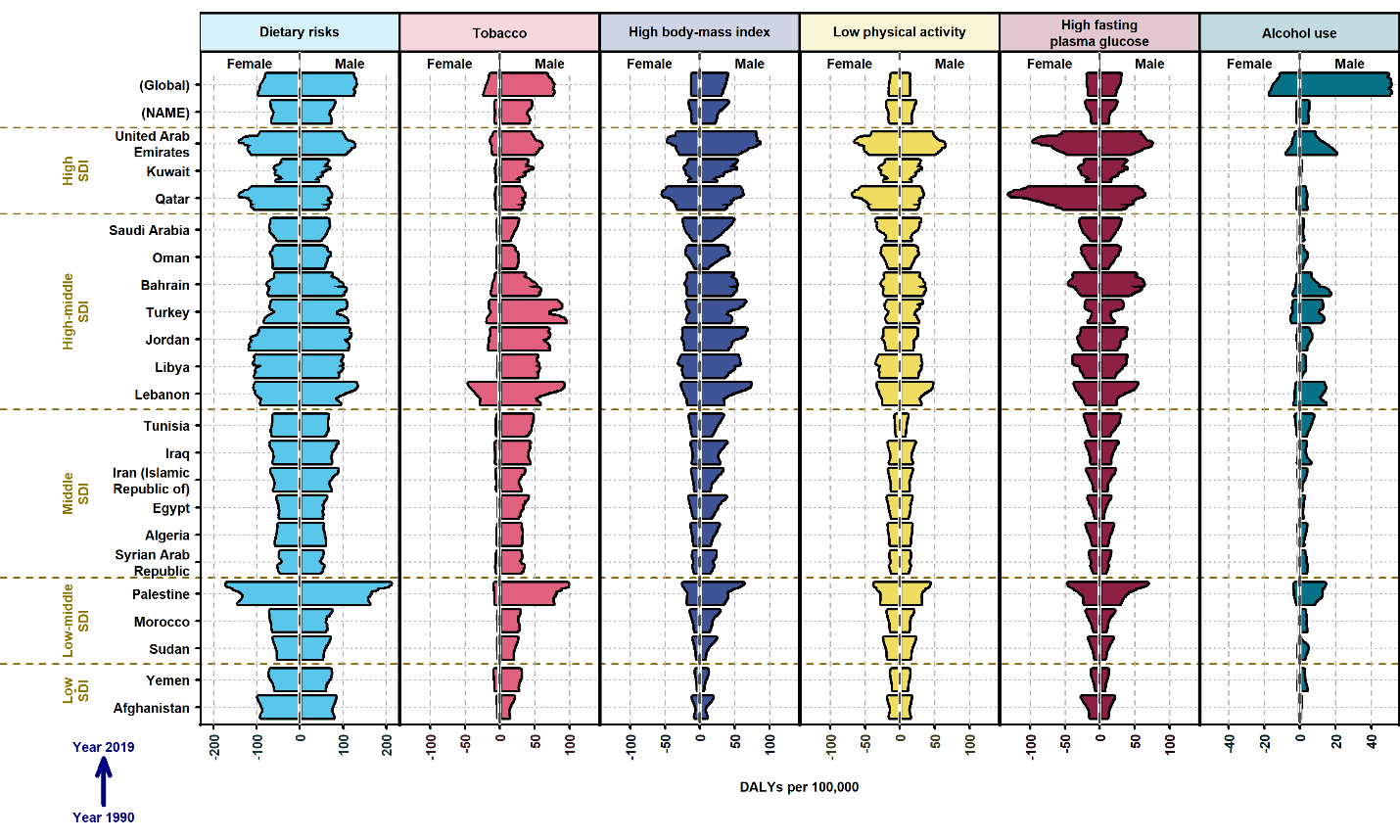
**

**Supplementary Figure 4.** National trends of colorectal cancer DALYs attributed to risks factors by gender. DALYs, disability adjusted life years.

**
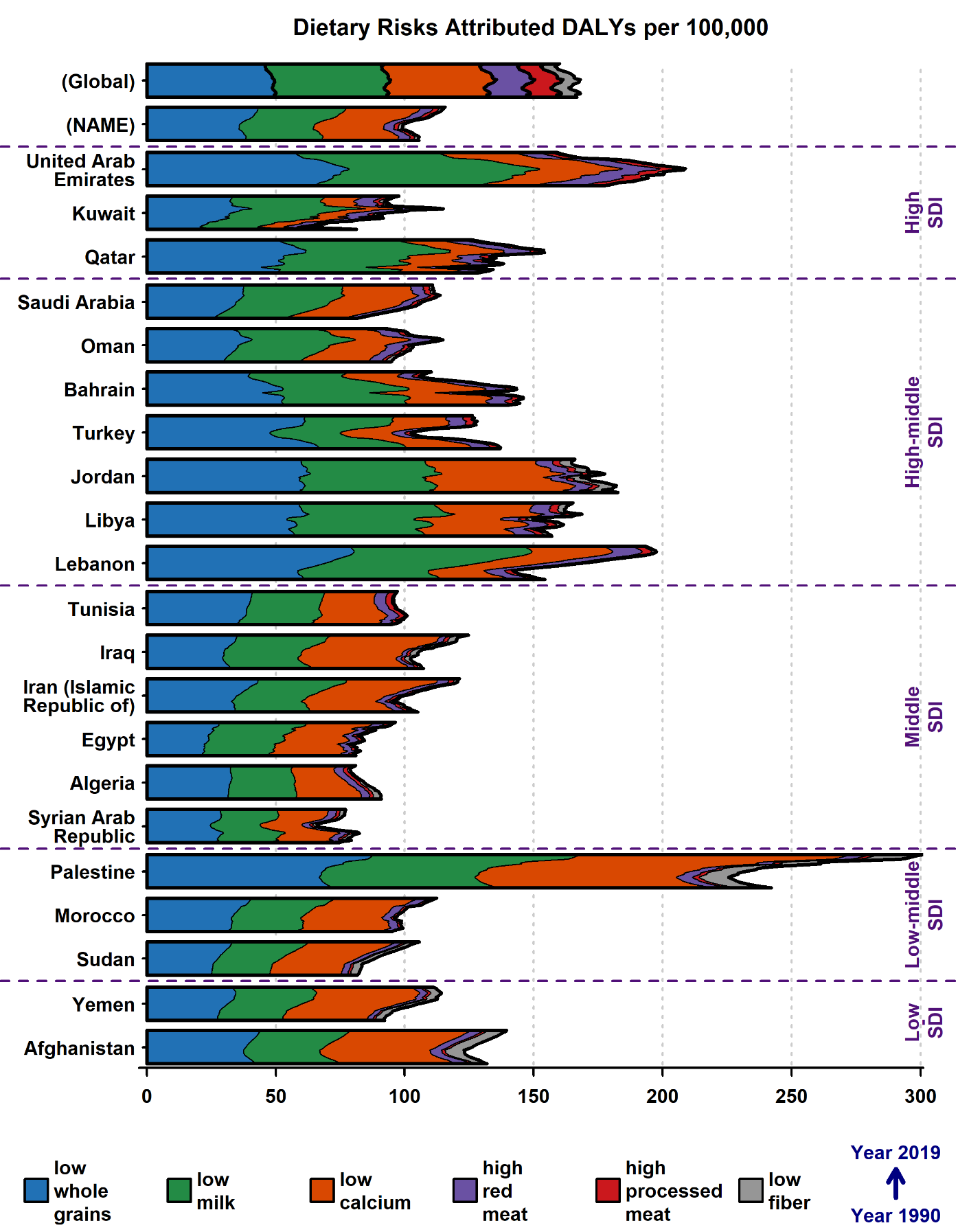
**

**Supplementary Figure 5.** National trends of colorectal cancer DALYs attributed to dietary risks factors by gender. DALYs, disability adjusted life years.

**
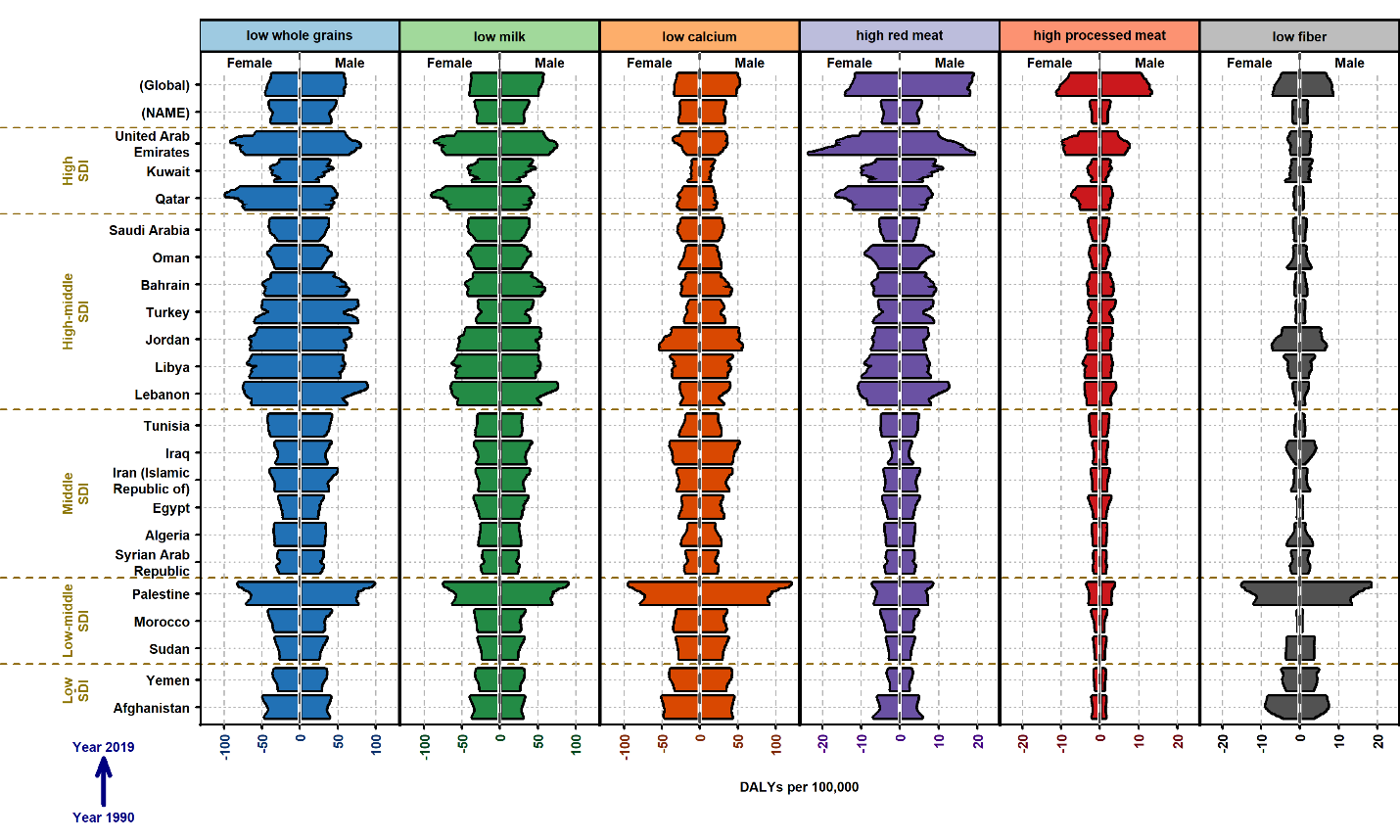
**

**Supplementary Figure 6.** National trends of colorectal cancer DALYs attributed to dietary risks factors by gender. DALYs, disability adjusted life years.
